# BiVO_4_–Cu_2_O/CuO Nanocubes with High Charge Injection and Charge Separation Rates for Enhanced Photoelectrochemical Water Oxidation

**DOI:** 10.1021/acsaem.5c02177

**Published:** 2025-10-30

**Authors:** Suzanne M.E. Assen, Willemijn H. Boeije, Pieter de Haij, Camilo A. Mesa, Ana Gutiérrez-Blanco, Laura Montañés, Sixto Giménez, Huub J.M. de Groot

**Affiliations:** a Leiden Institute of Chemistry, 4496 Leiden University, Einsteinweg 55, Leiden 2300 RA, The Netherlands; b Institute of Advanced Materials (INAM), 16748 Universitat Jaume I, Avenida de Vicent Sos Baynat, s/n, Castellón de la Plana 12006, Spain

**Keywords:** photoanode, bismuth vanadate, oxygen evolution, copper oxide, nanocubes, nanowires, charge separation efficiency, charge injection efficiency

## Abstract

Bismuth vanadate (BiVO_4_) shows promise as a photoanode for water oxidation, with a relatively low band gap of 2.4 eV. However, its performance is limited by poor carrier separation and surface recombination. To address these limitations, a BiVO_4_–Cu_2_O/CuO nanocube (NC) heterojunction was successfully developed, increasing the photogenerated oxygen evolution current density to 2.3 mA/cm^2^ at 1.23 V vs RHE, compared to 1.4 mA/cm^2^ for bare BiVO_4_. Addition of drop-casted Cu_2_O/CuO NCs on BiVO_4_ increases both the charge injection efficiency and the charge separation efficiency to over 60% at 1.23 V vs RHE. In comparison, the addition of drop-casted CuO nanowires (NWs) to BiVO_4_ increased the charge injection efficiency to over 65%, but it only moderately increased the charge separation efficiency to 49% at 1.23 V vs RHE. These results highlight the importance of the interface between BiVO_4_ and the catalyst layer for an enhanced photocurrent. In the Cu_2_O/CuO NCs, bulk Cu_2_O likely serves as a hole-extracting heterojunction, while surface CuO likely functions as a catalyst. In contrast, the CuO NWs function primarily as a catalyst.

## Graphical abstract

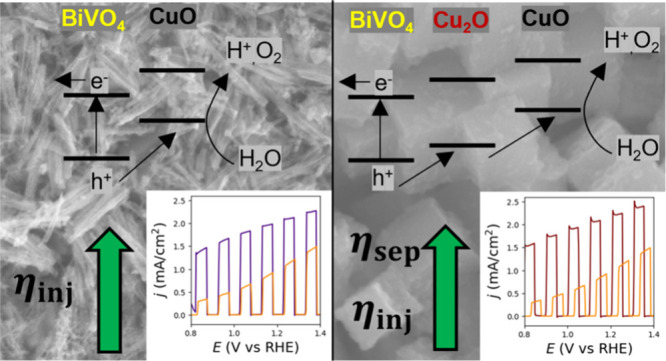

## Introduction

Photoelectrochemical (PEC) water splitting is an emerging technology to convert solar energy into storable hydrogen or carbon energy.^1,2^ In a PEC system, a photoanode can be combined with a cathode or a photocathode to facilitate hydrogen production, CO_2_ reduction into carbon-based products, or other reduction reactions.^3–5^ Among photoanode materials, bismuth vanadate (BiVO_4_), generally an n-type semiconductor with a band gap of 2.4 eV, attracted attention due to its potential to theoretically generate up to 7.5 mA/cm^2^, outperforming commonly studied photoelectrochemical materials such as TiO_2_ and WO_3_.^6–10^ However, the performance of BiVO_4_ is hampered by poor charge separation, by surface recombination effects, and by photocorrosion.^8,10^ To overcome these hurdles, several strategies have been explored, such as nanostructuring,^11^ facet-engineering,^12,13^ doping,^14,15^ creating a heterojunction,^16–19^ or depositing a cocatalyst,^19–24^ all of which have demonstrated enhanced photoelectrochemical water oxidation. In general, integrating two semiconductors in a Type II heterojunction can enhance the PEC performance, for example improving the charge separation efficiency (η_sep_), facilitating efficient hole transfer from the cocatalyst to the water, or increasing the charge injection efficiency (η_inj_).^18,25,26^ In contrast, a cocatalyst on BiVO_4_ can help prevent recombination via the surface states and enhance the water oxidation kinetics, improving η_inj_.^20,21^ For improving the photocurrent of BiVO_4_, the use of earth-abundant materials is desirable.^27^

Here, we aim to construct a type II heterojunction for water oxidation by combining BiVO_4_ with CuO. Copper oxide is a widely available, nontoxic material well-suited for electrochemical water oxidation applications.^27–30^ CuO is a p-type semiconductor with a band gap ranging from 1.2 to 1.7 eV, depending on the preparation method.^18,31–33^ Many CuO nanostructures with different morphologies have been synthesized.^27,34^ In addition, CuO and Cu_2_O can be used as cathodic CO_2_ reduction catalysts or photocathodes in combination with other semiconductors when a negative bias is applied.^5,35,36^

Nanostructured copper oxide can be a very rapid OER catalyst under a positive bias voltage.^28^ High catalytic rates have been attributed to a characteristic vibrational structure with collective oxygen modes along distorted quasi-1D CuO chains that facilitate catalysis through electron spin alignment for the O–O bond formation.^28^ Although few studies have examined CuO as an active catalyst in combination with BiVO_4_ for the OER, previous studies have shown that combining CuO or Cu_*x*_O with BiVO_4_ can improve the η_inj_ significantly.^18,31,37^ Furthermore, a Cu_2_O layer on BiVO_4_ can function as a hole-extracting layer, often in combination with another catalyst (Table S1).^30,31,38,39^ We investigated the combination of BiVO_4_ with two different nanostructured copper oxides, using either Cu­(OH)_2_ or Cu_2_O as a precursor. Our data corroborate the earlier findings and provide additional evidence that distortion of the structure in spherical morphologies can help facilitate catalysis, as well as Cu_2_O serving as a hole-extracting layer.^28,30,31,38,39^

This work uses the drop-casting method to combine copper oxide nanoparticles with BiVO_4_.^31,33,40,41^ The addition of CuO nanowires (NW), derived from Cu­(OH)_2_, to BiVO_4_ results in a favorable η_inj_, leading to a significant improvement in the photoresponse of 2.1 mA/cm^2^ compared to 1.4 mA/cm^2^ for bare BiVO_4_ at 1.23 V vs RHE, indicating that the CuO NWs effectively function as a cocatalyst. Further improvement is obtained with Cu_2_O/CuO nanocubes (NCs) instead of CuO NWs, which are derived from Cu_2_O. With NCs on BiVO_4_, the photogenerated current density reaches 2.3 mA/cm^2^ at 1.23 V vs RHE. Both the CuO NW and the Cu_2_O/CuO NC show small spherical particles on their respective surfaces consisting predominantly of CuO, which can contribute to facilitating catalysis by enhancing η_inj_ relative to undistorted morphologies.^28^ Although the BiVO_4_–CuO NW combination displays higher η_inj_ than BiVO_4_–Cu_2_O/CuO NC, the combination of enhanced η_sep_ and η_inj_ of the BiVO_4_–Cu_2_O/CuO NC provides higher photogenerated current densities, compared to both BiVO_4_–CuO NWs and bare BiVO_4_, indicating that Cu_2_O/CuO NCs both form a heterojunction to aid the charge separation and function as a cocatalyst for increased charge injection when drop-casted on BiVO_4_.

## Experimental Section

### Materials

Copper nitrate trihydrate (Cu­(NO_3_)_2_·3H_2_O), sodium hydroxide (NaOH), l-ascorbic acid (AA, C_6_H_8_O_6_), Nafion (5 wt %), sodium dodecyl benzenesulfonate (SDBS, C_18_H_29_NaO_3_S), copper chloride (CuCl_2_), sodium sulfite (Na_2_SO_3_), sodium carbonate (Na_2_CO_3_), sodium bicarbonate (NaHCO_3_), sodium hydroxide (NaOH), potassium phosphate (KH_2_PO_4_), dipotassium phosphate (K_2_HPO_4_), potassium carbonate (K_2_CO_3_), potassium bicarbonate (KHCO_3_), boric acid (H_3_BO_3_), potassium hydroxide (KOH), Bismuth­(III) nitrate (Bi­(NO_3_)_3_), Potassium iodide (KI), lactic acid (C_3_H_6_O), nitric acid (HNO_3_), *p*-benzoquinone (C_6_H_4_O_2_), and vanadylacetylacetonaat (VO­(acac)_2_) were obtained from Sigma-Aldrich.

### Synthesis of BiVO_4_ Films

BiVO_4_ films are prepared by a modified previous reported method.^42^ Before the film deposition, fluorine-doped tin oxide (FTO) substrates are washed ultrasonically in soap water (Extran, Sigma-Aldrich), Milli-Q water, and a mixture of acetone/isopropanol (1:3) each for 14 min. Subsequently, rinsed FTO substrates are cleaned by using a UV ozone chamber right before their use. First, the plating solution is prepared by adding 0.02 mol of Bi­(NO_3_)_3_ in 50 mL of a 0.4 M KI solution containing 0.06 M lactic acid at a pH 1.7 adjusted by HNO_3_. Then, 20 mL of a 0.23 M *p*-benzoquinone solution is slowly added into the plating solution under stirring. A three-electrode system is used for electrodeposition. A platinum sheet counter electrode (CE), a Ag/AgCl (3 M KCl) reference electrode (RE), and an FTO substrate (WE) are used. The electrochemical deposition was performed in two steps. First, a potential of −0.35 V vs Ag/AgCl during 20 s is applied for the nucleation step. The growing step was performed by applying −0.1 V for 300 s at room temperature, which was equivalent to passing a charge of −0.19 C/cm^2^. The precipitation of BiOI onto FTO is produced by the increase of the local pH on the WE due to the reduction of *p*-benzoquinone to hydroquinone by applying a cathodic bias. The BiOI films are subsequently washed with milli-Q water and air-dried. Onto BiOI, 50 μL of 0.2 M VO­(acac)_2_ solution in DMSO is deposited by drop-casting in a heating plate at 80 °C. The deposited films are calcined at 475 °C (heating rate, 2 °C/min) for 1 h. The excess V_2_O_5_ is removed by soaking the photoanodes in a 1 M NaOH solution under vigorous stirring for 10 min followed by washing with Milli-Q water and air-drying.

### Synthesis of CuO NWs

CuO nanowires (NWs) are synthesized via an adapted method from Ma et al.^33,41^ Initially, 0.02 g of SDBS is added to 50 mL of 12.5 mM CuCl_2_ under continuous stirring. After 10 min of stirring, 50 mL of 12.5 mM K_2_CO_3_ is added dropwise to the solution, which is then stirred for at least 30 min. Subsequently, 2.0 g of NaOH is added to the solution under vigorous stirring, and the mixture is stirred for another 10 min. Next, the mixture is ultrasonicated for 20 min and centrifuged down. The resulting blue precipitate is washed three times with water and three times with ethanol and then dried overnight at 60 °C to yield a blue Cu­(OH)_2_ NW powder. Finally, the powder is calcined for 2 h at 300 °C to produce the black CuO NW powder (Figure S1a).

### Synthesis of Cu_2_O/CuO NCs

Cu_2_O/CuO nanocubes (NCs) are synthesized using an adapted method from Huang et al.^40^ Initially, 16 mL of 0.113 M NaOH is added to 16 mL of 5 mM Cu­(NO_3_)_2_·3H_2_O and stirred thoroughly. Next, 10 mL of 9 mM ascorbic acid is added dropwise under continuous stirring. The mixture is then stirred for at least 30 min until an orange turbid mixture is obtained. This mixture is then centrifuged and washed three times with water and three times with ethanol. The precipitate is dried at 60 °C until a dry orange-red Cu_2_O NC powder is obtained. Finally, the powder is calcined at 300 °C for 3 h to yield the black Cu_2_O/CuO NC powder (Figure S1b).

### Preparation of the BiVO_4_–CuO NW and BiVO_4_–Cu_2_O/CuO NC (Photo)­anodes

The prepared BiVO_4_ film is rinsed with Milli-Q (MQ) water before the copper oxide powder is drop-casted onto the surface. For the measurements without BiVO_4_, FTO electrodes obtained from Sigma-Aldrich are cut into 2.5 cm × 1 cm pieces. The FTO plates are sonicated in acetone for 20 min and thoroughly rinsed with MQ water. The obtained copper oxide (NC/NW) powder is ground using a mortar until a fine powder is obtained. For the CuO NW, a suspension is prepared by mixing 10 mg of CuO NW with 450 μL of ethanol and 25 μL of 5 wt % Nafion, which is ultrasonicated for 20 min to produce a catalyst ink. Of this catalyst ink, 30 μL/cm^2^ is drop-casted onto the substrates and allowed to dry for at least an hour. For the Cu_2_O/CuO NC, a catalyst ink is prepared by creating a suspension with 6 mg of CuO NC, 591 μL of water, 394 mL of ethanol, and 15 μL of 5 wt % Nafion. The catalyst ink is ultrasonicated for 20 min, after which 100 μL/cm^2^ is then drop-casted onto the earlier prepared BiVO_4_ or FTO substrates and dried overnight. (Figure S1c)

### Characterization

To determine the morphology of the prepared samples, scanning electron microscopy (SEM) was performed with a Thermo Fisher Apreo SEM microscope. In addition, SEM with energy-dispersive X-ray spectroscopy (SEM-EDX) was used to determine the elemental composition of the electrodes. To determine the crystal structure, X-ray diffraction (XRD) measurements of the electrodes were performed with a Philips X’Pert diffractometer in a Bragg–Brentano geometry, equipped with an X’Celerator detector and a Cu–Kα source. The crystallite size *D* of the synthesized particles is estimated using the Scherrer equation

$$D=\frac{K\lambda }{B\cos\left(\theta \right)}$$ 1

where *K* = 0.9 is the Scherrer constant, λ = 1.54 Å is the wavelength of the X-ray beam used, *B* is the full width at half-maximum (fwhm) of the peak in radians, and θ is the Bragg angle in radians.^43^

Deposited Cu materials were analyzed with a NAPXPS system using a 30 W SPECS Al Kα lab X-ray source (μFOCUS 600 Monochromatic X-ray Source, *E* = 1486.7 eV) and Phoibos 150 analyzer unit (SPECS).^44^ The *XY* position of the sample was adjusted by using count rates of Cu 2p_3/2_ before analyzing the samples.

The light absorption data for the BiVO_4_ electrodes were retrieved from the UV–vis measurements on a Lambda 1050+ spectrophotometer (PerkinElmer) with an integrating sphere using BaSO_4_ as reference. The absorbance (*A*) was estimated by *A* = −log­(*T* + *R*)/100, where *T* is the transmittance and *R* is the diffuse reflectance.

Incident photon to current efficiency (IPCE) measurements were carried out using an ozone-free 300 W Xe lamp combined with a monochromator (Oriel Cornerstone 130, 1/8 m) and an optical power meter.

### (Photo)­electrochemical Measurements

All glassware was cleaned by overnight immersion in a KMnO_4_ solution and subsequently rinsed with MQ water at least 3 times. Next, the glassware was submerged in a diluted hydrogen peroxide and sulfuric acid solution followed by another rinse with MQ water. Finally, the glassware was boiled three times in MQ water. The (photo)­electrochemical cyclic voltammetry (CV) and linear sweep voltammetry (LSV) measurements were conducted at room temperature using an Autolab PG-stat10 potentiostat with a stepsize of 2.5 mV. The instrument is equipped with a FRA32 M module for electrochemical impedance spectroscopy (EIS). A homemade quartz glass container was used, with the prepared samples as the working electrode (WE), a platinum wire from Mateck as the counter electrode (CE), and a reversible hydrogen electrode (RHE) from Gaskatel as the reference electrode. Electrolytes were purged with argon gas (Linde Gas) for a minimum of 20 min prior to measurements. A solar simulator (SS-F5–3A) from Enlitech, equipped with an AM1.5G filter and calibrated to deliver *P* = 1000W/m^2^ (1 sun), was used to provide (chopped) illumination to the photoelectrode in an otherwise completely dark environment (Figure S2).

For measurements without light, the *iR* drop was compensated. First, an uncompensated resistance *R* = 20 Ω was measured by using the positive feedback module in the Metrohm Autolab Nova software. The voltage was subsequently corrected after the measurement according to^45^

$$E=E_{\mathrm{applied}}-j\cdot R$$ 2

For measurements with continuous or chopped illumination, the *iR* drop was neglected, as it is much smaller than for the dark measurements due to low current densities and decreased resistance of *R* ≤ 10 Ω under illumination. The potassium phosphate buffer (KPi) was prepared by adding K_2_HPO_4_ to KH_2_PO_3_ until pH 7 was obtained. Similarly, pH 10 potassium carbonate buffer (KCi) was prepared by mixing KHCO_3_ with K_2_CO_3_. The potassium borate buffer (KBi) was prepared by adding 1 M KOH to H_3_BO_3_ to obtain pH 9.5. The pH of the electrolytes was determined with a Consort P901 pH meter.

### Photoelectrochemical Parameter Calculations

The total photogenerated current density of a photoanode during the oxygen evolution reaction (*j*_OER_) can be described as

$$j_{\mathrm{OER}}=j_{\mathrm{abs}}\cdot \eta_{\mathrm{sep}}\cdot \eta_{\mathrm{inj}}$$ 3

where the theoretical maximum photogenerated current density of the photoanodes (*j*_abs_) can be estimated based on the measured absorption spectrum in conjunction with the AM1.5G spectrum (Figure S3).^46,47^

The applied bias photon-to-current efficiency (ABPE) can be calculated by

$$\mathrm{ABPE}=\frac{j_{\mathrm{OER}}\cdot \left(1.23V-E\right)}{P}\cdot 100\%$$ 4

Using the assumption that the charge injection efficiency η_inj_ ≈ 1 when 0.5 M Na_2_SO_3_ is added to the electrolyte to act as a hole scavenger, we can use eq 3 to estimate the charge separation efficiency

$$\eta_{\mathrm{sep}}\approx \frac{j_{{\mathrm{Na}}_{2}{\mathrm{SO}}_{3}}}{j_{\mathrm{abs}}}$$ 5

where *j*_Na_2_SO_3__ represents the measured photogenerated current density with 0.5 M Na_2_SO_3_ acting as a hole scavenger. In addition, we estimate

$$\eta_{\mathrm{inj}}\approx \frac{j_{\mathrm{ph}}}{j_{{\mathrm{Na}}_{2}{\mathrm{SO}}_{3}}}$$ 6

where *j*_ph_ is the measured photogenerated current density without a hole scavenger present.^47^

The hole relaxation lifetime (τ_p_) is determined from the Bode phase plot by

$$\tau_{p}=1/2\pi f$$ 7

where *f* is the frequency where the Bode phase is at its maximum.^48^

The IPCE is calculated by

$$\mathrm{IPCE}=\frac{1240\cdot j_{\mathrm{ph}}}{\lambda \cdot P}$$ 8

where *j*_ph_ is the measured photocurrent density, λ is the incident wavelength, and *P* is the power of monochromatic light at that wavelength.^49^

Measurements were repeated to ensure reproducibility. Error values are reported when they differ beyond the last decimal place.

## Results and Discussion

Figure 1a presents an SEM image of a BiVO_4_ film, revealing BiVO_4_ nanoparticles with uniform coverage of the FTO substrate. Figure 1d provides a magnified view of the nanoparticles, with rounded shapes and a size distribution between 80 and 150 nm, in line with data reported by Arcas et al.^50^ Figure 1b shows the prepared CuO NW deposited on top of a BiVO_4_ layer, with an enlarged view provided in Figure 1e. The NWs exhibit mostly a random orientation within the plane parallel to the BiVO_4_ and FTO layers. They are approximately 1000 nm in length and 30 nm in width. This is slightly larger than the nanowires prepared by Ma et al. and with a similar aspect ratio of over 20.^33^ Compared to the Cu­(OH)_2_ NW precursor (Figure S4), the CuO NWs exhibit an additional structure, with spherical particles of about 10–20 nm forming after calcination (Figure 1e), indicating lattice defects.^34,51^ Figure 1c shows the prepared Cu_2_O/CuO NCs deposited on top of a BiVO_4_ layer, with an enlargement provided in Figure 1f. The Cu_2_O/CuO NCs have an edge length of approximately 400–700 nm, are randomly oriented, and cover the BiVO_4_ layer. The precursor, Cu_2_O NC, shows well-defined cubes (Figure S5). There are small, spherical particles ranging from 10 to 50 nm on the surface of the Cu_2_O/CuO NCs, consistent with the results of Huang et al.^40^ The spherical motifs on the surface appear after the calcination process, during which Cu_2_O was partly oxidized into CuO. Higher-magnification SEM is shown in Figures S6a,b. A side-view SEM image (Figure S6c) confirms that the NCs rest on top of the BiVO_4_. An EDX spectrum taken near the edge of a BiVO_4_–Cu_2_O/CuO NC sample (Figure S7), where the Cu_2_O/CuO NC coverage is sparse, shows the detection of Cu at the cubes, while Bi, V, and Sn are primarily detected in the area without any cubes. Oxygen is found across the entire sample. Photographs of the front and back of the prepared samples (Figure S8) demonstrate that the illuminated FTO/BiVO_4_ interface side exhibits the characteristic yellow of BiVO_4_, while the BiVO_4_/copper oxide/electrolyte interface side shows the black of the copper oxide powders, indicating that the catalyst ink can be drop-casted without damaging the underlying BiVO_4_ layer. Figure 1g shows the measured XRD patterns of BiVO_4_ as well as BiVO_4_ in combination with Cu_2_O/CuO NCs or CuO NWs, with a closeup of the spectra in Figure 1h. The XRD pattern of the FTO-BiVO_4_ sample aligns well with the reference spectra. Using the Scherrer equation (eq 1), the estimated crystallite sizes of the BiVO_4_ range from ca. 40 to 60 nm (Table S2), slightly smaller than the particle size observed with the SEM, suggesting their polycrystalline nature. The FTO-BiVO_4_–CuO NW diffraction data in Figure 1g display peaks that can be attributed to SnO_2_, BiVO_4_, and CuO. Figure S9 shows the XRD spectra of the FTO-BiVO_4_–CuO NW sample and its parent materials, FTO, BiVO_4_, CuO NWs, and the intermediate Cu­(OH)_2_ NW. The Cu­(OH)_2_ NW precursor diffraction pattern can be associated with reflections from the Cu­(OH)_2_ (020), (021), (002), (111), (022), (130), and (132) lattice planes. Upon calcination, the diffraction pattern can be associated with CuO (002)/(1̅11), (022)/(111), and (113) reflections, with the diffraction peak associated with (002)/(1̅11) slightly higher than the diffraction peak associated with (022)/(111), corresponding to the monoclinic space group *C*1*c*1.^52^ In the CuO NW pattern, Cu­(OH)_2_ diffraction appears suppressed. The FTO-BiVO_4_–Cu_2_O/CuO NC XRD spectrum shows, in addition to the diffraction from FTO-BiVO_4_, patterns matching the Cu_2_O and CuO phases (Figure 1g). This suggests that the calcination process produces a mix of Cu_2_O and CuO. This contrasts with the study of Huang et al., who focused on complete conversion of Cu_2_O to CuO after calcination.^40^ Figure S10 shows the FTO-BiVO_4_–Cu_2_O/CuO NC sample on the FTO substrate with its parent materials FTO, BiVO_4_, and Cu_2_O/CuO NC and the intermediate Cu_2_O NC. Diffraction associated with the Cu_2_O (110), (111), (200), and (220) lattice planes are visible in the Cu_2_O NC precursor and the Cu_2_O/CuO NC sample, albeit with reduced intensity in the Cu_2_O/CuO NC compared to that in the Cu_2_O NC sample in its cubic space group Pn3̅m .^53^ After calcination, diffraction responses from the (002)/(1̅11) and (022)/(111) lattice planes of CuO in the monoclinic space group appear, confirming a partial transformation of Cu_2_O to CuO.^52^ Figure S11 shows XRD spectra of CuO NW, Cu­(OH)_2_ NW, Cu_2_O/CuO NC, and Cu_2_O NC powders. Based on the diffraction patterns in Figure S11 and the Scherrer equation (eq 1), the crystallinity size of the CuO NW is ca. 7–12 nm, similar to its precursor Cu­(OH)_2_ NW (Table S2). For the Cu_2_O/CuO NC sample, the CuO crystallite size is estimated at ca. 10–14 nm, while the Cu_2_O retains a size estimate of *ca*. 30–40 nm, for both the final Cu_2_O/CuO NC and its precursor form (Table S2). A schematic overview of the layers of the photoanode is presented in Figure 1i, starting from a glass layer, followed by an FTO layer, a BiVO_4_ layer, and a CuO NW or Cu_2_O/CuO NC layer, where the water oxidation takes place. XPS analysis of the FTO-CuO NW and FTO-Cu_2_O/CuO NC (Figure S12) reveals highly similar spectral features, indicating comparable surface compositions. The full survey spectrum (Figure S12a) displays, among others, characteristic signals of O 1s, Cu 2p, Cu 3s, and Cu 3p.^54^ In the Cu 2p region (Figure S12b), pronounced peaks at 953 eV (Cu 2p_1_/_2_) and 933 eV (Cu 2p_3_/_2_), together with strong shakeup satellites at 943, 951, and 962 eV, are indicative of Cu^2+^ species. The Cu LMM Auger spectrum (Figure S12c) shows a kinetic energy maximum at ∼918.1 eV and the O 1s peak at ∼529.3 eV (Figure S12d), indicating the presence of CuO as the dominant surface phase for both samples.^40,55^ Finally, the valence band spectrum (Figure S12e) reveals that the Fermi level is located near the valence band maximum, in agreement with the p-type semiconducting character of CuO.^56^

**Figure 1:**
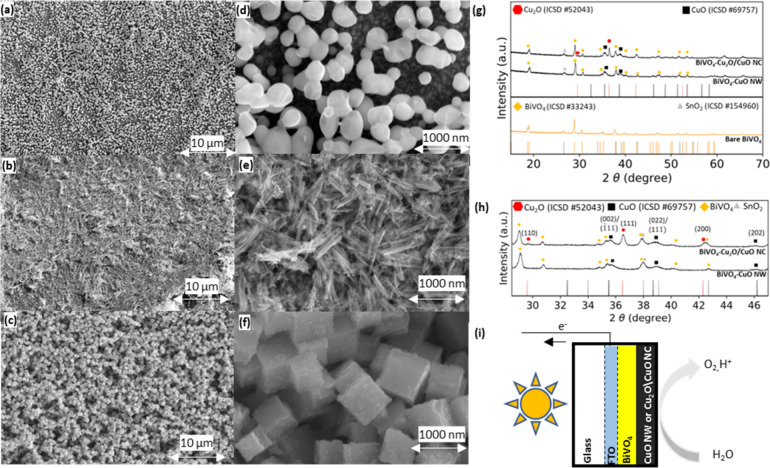
SEM images of overview and closeup of (a, d) pure BiVO_4_ on FTO, (b, e) CuO NWs on BiVO_4_, and (c, f) Cu_2_O/CuO NCs on BiVO_4_. (g) XRD spectra over a broad spectrum for BiVO_4_ with and without Cu_2_O/CuO NC or CuO NW particles. (h) A closeup of the XRD spectra of BiVO_4_–CuO NW and BiVO_4_–Cu_2_O/CuO NC samples. The samples are compared to ICSD reference spectra for SnO_2_, BiVO_4_, Cu_2_O, and CuO with collection codes 154960, 33243, 52043, and 69757, respectively. (i) Schematic overview of layers of the sample.

Figure 2a shows CV data collected in the dark from the CuO NW and Cu_2_O/CuO NC on FTO in 1 M KCi pH10 buffer solution. The onset potential for the OER catalysis is observed around ca. 1.77 V vs RHE for the Cu_2_O/CuO NC catalyst and around ca. 1.70 V vs RHE for the CuO NW catalyst. Figure 2b shows that consistent with the findings of Liu et al.,^41^ the CuO NW exhibits lower catalytic onset potentials at increasing pH values. In a 1 M pH 7 KPi buffer, the onset is found at ca. 1.85 V vs RHE, while it is around ca. 1.70 V vs RHE in a 1 M pH 9.5 KBi buffer or a 1 M pH 10 KCi buffer. The CuO NW has a lower Tafel slope (138 ± 5 mV/dec) in the KCi buffer compared to the KBi buffer (182 ± 5 mV/dec, inset Figure 2b).^57^ The CuO NW is very unstable in a 1 M KOH buffer; however, in the first scan, it is possible to detect an onset potential of *ca*. 1.60 V vs RHE, which is the lowest found for this system. The detailed pH-dependent plot in Figure S13 shows the catalyst response between pH 7 and pH 14. The Tafel slope of the CuO NW appears to be independent of pH with values between 140 and 250 mV/dec (Figure S13c). If we exclude the low onset potentials for 0.1 and 1 M KOH, where Cu­(OH)_4_^2–^ can form according to the Pourbaix diagram,^58^ the onset potentials suggest near-Nernstian behavior.^45^ Figure 2c shows that a similar trend can be observed for the Cu_2_O/CuO NCs, with the lowest onset potential of ca. 1.7 V vs RHE in 1 M KOH with a Tafel slope of 160 ± 5 mV/dec. The Tafel slope in the pH 10 KCi buffer was 141 ± 5 mV/dec, compared to 178 ± 5 mV/dec in the pH 9.5 KBi buffer (inset in Figure 2c). For the Cu_2_O/CuO NCs, the smallest Tafel slope of 110 ± 5 mV/dec is observed at pH 10.5 in a NaCi buffer (Figure S14c), while Tafel slopes exceed 200 mV/dec for pH below 9 and rise over 150 mV/dec for pH over 11. At both pH 9.5 and 10, the Tafel slope is lower in a NaCi buffer than that in a NaBi buffer. The onset potentials at various pH (Figure S14d), again excluding the 0.1 and 1 M KOH measurements, show near-Nernstian behavior.^45^ For both CuO NW and Cu_2_O/CuO NC in KOH buffers, it was visually observed that the catalyst powders detached from the FTO during measurements, in line with the formation of Cu­(OH)_4_^2–^.^58^ Figure S15a compares the CuO NW and Cu_2_O/CuO NC with their precursors. The Cu_2_O/CuO NC and CuO NW samples show increased current densities compared with their respective precursors. Figure S15b,c indicates that both the CuO NWs and Cu_2_O/CuO NCs show an increase in the Tafel slopes when the molarity of the solution increases, likely due to improved mass transport characteristics.^57^ At 1.5 M KCi, the samples showed the best OER activity but were highly unstable. To allow for multiple consecutive scans, it is necessary to perform experiments at a concentration of ≤1 M KCi.

**Figure 2:**
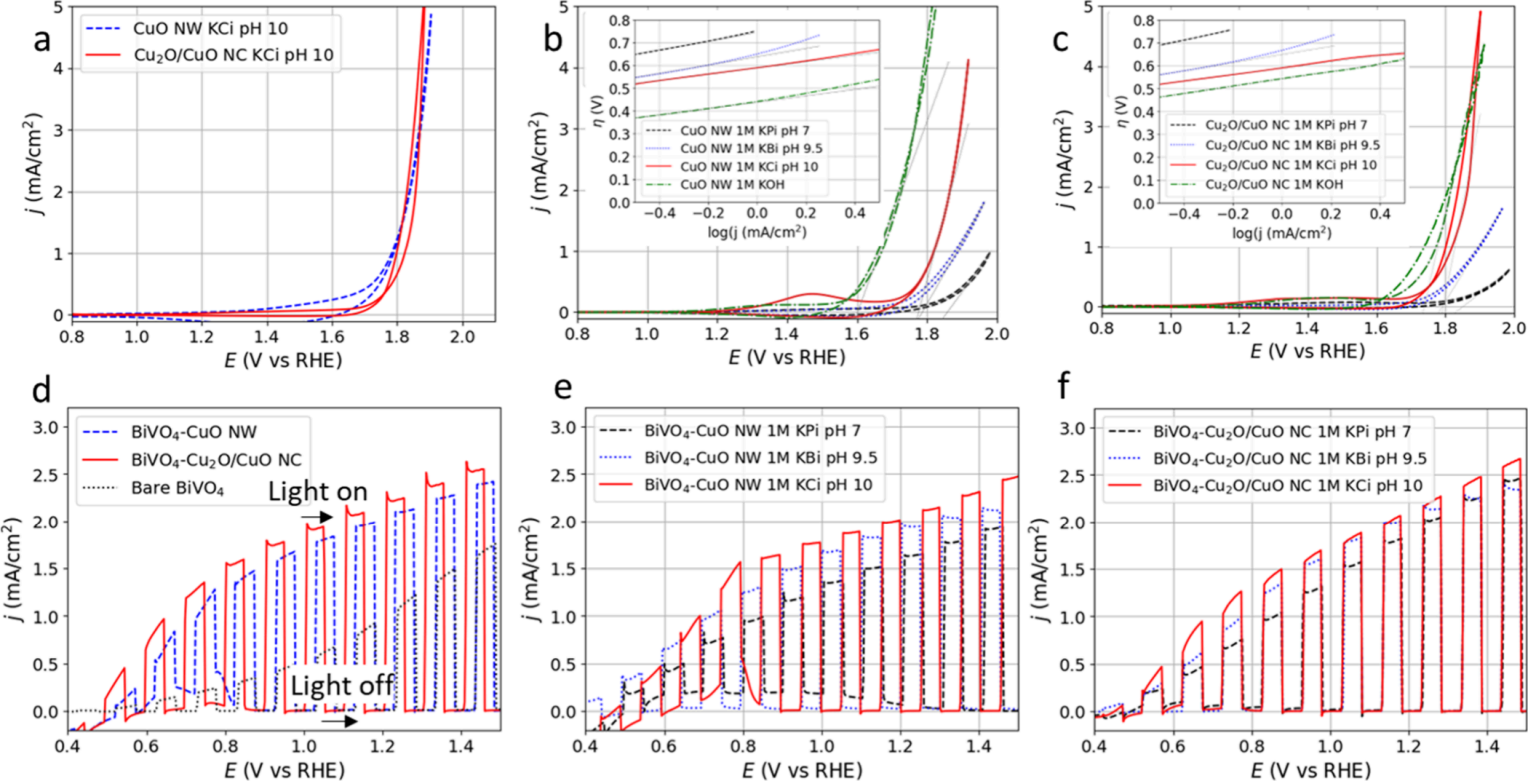
CV curves and LSV curves of the samples. (a) Characteristic dark CV curves of the CuO NW or Cu_2_O/CuO NC on FTO using 1 M KCi pH 10 buffer. Dark CV curves of (b) the CuO NW or (c) Cu_2_O/CuO NC for different buffers with the Tafel plot as an insert. All dark curves are corrected with 20 Ω resistance. (d) LSV curves of BiVO_4_ with and without the CuO NW or Cu_2_O/CuO NC using a 1 M KCi pH 10 buffer under chopped AM1.5G illumination. LSV curves of a single (e) BiVO_4_–CuO NW and (f) BiVO_4_–Cu_2_O/CuO NC measured in different buffers with increasing pH. For the CV data with chopped illumination, internal resistance effects are considered negligible and uncorrected data are shown. The scan rate is 10 mV/s.

The chopped illuminated LSV curves in Figure 2d show that drop-casting the CuO NW or Cu_2_O/CuO NC on top of BiVO_4_ significantly enhances the photogenerated OER activity of the BiVO_4_. The BiVO_4_–Cu_2_O/CuO NC combination generates the highest *j*_ph_, at relatively low potentials. For example, at 0.85 V versus RHE, *j*_ph_ increases from 0.68 mA/cm^2^ for bare BiVO_4_ to 1.39 mA/cm^2^ for BiVO_4_–CuO NWs and 1.58 mA/cm^2^ for the BiVO_4_–Cu_2_O/CuO NC combination. At 1.23 V vs RHE, *j*_ph_ increases from 1.39 mA/cm^2^ for bare BiVO_4_ to 2.10 mA/cm^2^ for BiVO_4_–CuO NWs and 2.25 mA/cm^2^ for BiVO_4_–Cu_2_O/CuO NCs. The LSVs are converted into ABPE values using eq 4, indicating that BiVO_4_–CuO NWs, BiVO_4_–Cu_2_O/CuO NCs, and BiVO_4_ have maximum efficiencies of 0.58%, 0.66%, and 0.12%, respectively (Figure S16). Figure 2e shows a single BiVO_4_–CuO NW combination measured in different buffers with increasing pH levels. The best results are achieved in the pH 10 KCi buffer, with a clear improvement compared with the pH 7 KPi buffer. In the pH 10 KCi buffer for BiVO_4_–CuO NWs, a transient increase in current density is observed at 0.6–0.8 V vs RHE during the light-off periods, whereas in FTO-CuO NWs, measured in the dark in the pH 10 KCi buffer (Figure 2b), a comparable feature is located at 1.3–1.5 V vs RHE. The shift to lower bias in the presence of intermittent illumination and a BiVO_4_ interlayer indicates enhanced surface recombination.^59^ Additionally, in the pH 7 KPi buffer under intermittent illumination, the BiVO_4_–CuO NW displays an enhanced current between 0.5 and 1.3 V vs RHE with the light off, possibly with the same origin, while all current density for the pH 9.5 KBi buffer stems from illumination. Figure 2f presents the LSV curves for a single BiVO_4_–Cu_2_O/CuO NC combination measured in different buffers with increasing pH, where the pH 10 KCi buffer again yields the best results. In all buffers, the current density is observed upon illumination. For a single sample of bare BiVO_4_ (Figure S17), mostly similar *j*_ph_ is observed in a pH 8.5 or a pH 9.5 KBi buffer to that in a pH 10 KCi buffer, where *j*_ph_ rises from ∼0.1 mA/cm^2^ at 0.4 V vs RHE to 0.8–1.1 mA/cm^2^ at 1.23 V vs RHE.^60,61^ In an alkaline environment, the *j*_ph_ is higher than in a neutral pH 7 KPi buffer, where 0.5 mA/cm^2^ was reached at 1.23 V vs RHE. The CuO NW or the Cu_2_O/CuO NC in combination with BiVO_4_ exhibits an increased *j*_ph_ in the pH 10 KCi buffer over the full potential range compared to a pH 9.5 KBi and pH 7 KPi buffer (Figure 2e,f). This indicates that the CuO NW and the Cu_2_O/CuO NC, respectively, are likely the active catalysts, as they also displayed increased current densities in the dark under a pH 10 KCi buffer compared to a pH 9.5 KBi and pH 7 KPi buffer. Figure S18 demonstrates that the molarity of the buffer used influences the performance of all BiVO_4_ combinations. The bare BiVO_4_ and the BiVO_4_–CuO NW samples achieve optimal performance in a 0.5 M KCi buffer, whereas the BiVO_4_–Cu_2_O/CuO NC combination shows the best results in a 1 M KCi buffer. This behavior can be attributed to the Cu_2_O/CuO NC catalyst, which also shows the highest dark performance on FTO using 1 M KCi buffer (Figure S15c).

Figure 3a–c shows the LSV curves of BiVO_4_–CuO NWs, BiVO_4_–Cu_2_O/CuO NCs, and bare BiVO_4_ in pure buffer or with a 0.5 M NaSO_3_ hole scavenger. Figure S19 shows that *j*_Na_2_SO_3__ is the highest for BiVO_4_–Cu_2_O/CuO NCs at *E* > 0.8 V vs RHE, while the BiVO_4_–CuO NW slightly outperforms bare BiVO_4_. For the bare BiVO_4_ sample, a light response can be observed at 0.35 V vs RHE with the use of a hole scavenger, whereas it is observed around 0.55 V vs RHE during the OER. For the BiVO_4_–CuO NW and BiVO_4_–Cu_2_O/CuO NC combinations, the onset potential is similar, around 0.45 V vs RHE, regardless of the addition of a hole scavenger. Figure 3d shows the absorbance curve, with the midpoint of absorption estimated at ∼479 nm. The UV–vis data is converted into a Tauc plot, indicating that the band gap is 2.35 eV (inset Figure 3d), well in line with other reported BiVO_4_ combinations.^6–9,62^ Additionally, *j*_abs_ can be estimated at 4.95 ± 0.05 mA/cm^2^, assuming full photon absorption at the peak of the absorption curve in the AM1.5G spectrum (Figure S3).^46,63^ Using this *j*_abs_ value and *j*_Na_2_SO_3__for bare BiVO_4_, BiVO_4_–CuO NWs, and BiVO_4_–Cu_2_O/CuO NCs, η_sep_ was calculated according to eq 5 (Figure 3e). The BiVO_4_–Cu_2_O/CuO NC demonstrates a significant improvement in η_sep_ at *E* > 0.8 V vs RHE compared to bare BiVO_4_ with an increase from η_sep_ = 42% to η_sep_ = 67% at 1.23 V vs RHE. In contrast, the BiVO_4_–CuO NW combination displays only a slight improvement in η_sep_ at high potentials, with η_sep_ = 49% at 1.23 V vs RHE. Figure 3f shows that η_inj_, calculated according to eq 6, significantly improves after the addition of CuO NWs or Cu_2_O/CuO NCs on BiVO_4_, compared to bare BiVO_4_. For *E* > 1.0 V vs RHE, the BiVO_4_–CuO NW combination slightly outperforms the BiVO_4_–Cu_2_O/CuO NC combination. The most significant improvement in η_inj_ is observed at *E* of ∼0.8 V vs RHE, as η_inj_ < 20% for bare BiVO_4_ at this potential, while the combinations with copper oxide display a similar efficiency at higher potentials, with η_inj_ > 60%. The η_inj_ can be calculated up to *E* = 1.23 V vs RHE, since a significant dark response appears in the LSV with a hole scavenger at higher potentials. The combined effect of the improved η_sep_ and η_inj_ is most pronounced for the BiVO_4_–Cu_2_O/CuO NC combination, resulting in the highest photogenerated current compared to bare BiVO_4_ and BiVO_4_–CuO NWs (Figure 2d). A schematic overview of band alignments of BiVO_4_, Cu_2_O, and CuO is indicated in Figure 3g,h.^31^ Band gaps are based on Tauc plots for BiVO_4_ (inset of Figure 3d) and Cu_2_O (Figure S20b) and previous research for CuO.^33^ The UV–vis absorption spectrum for both the Cu_2_O NC and CuO NW (Figure S20a) appears to be compatible with this value. Band alignment is indicative, based on literature values in agreement with the observed increased charge separation for BiVO_4_–Cu_2_O/CuO NC compared to bare BiVO_4_ (Figure 3e) and the position of the valence band of CuO being close to the Fermi level by VB XPS (Figure S12e).^6,31,63^

**Figure 3:**
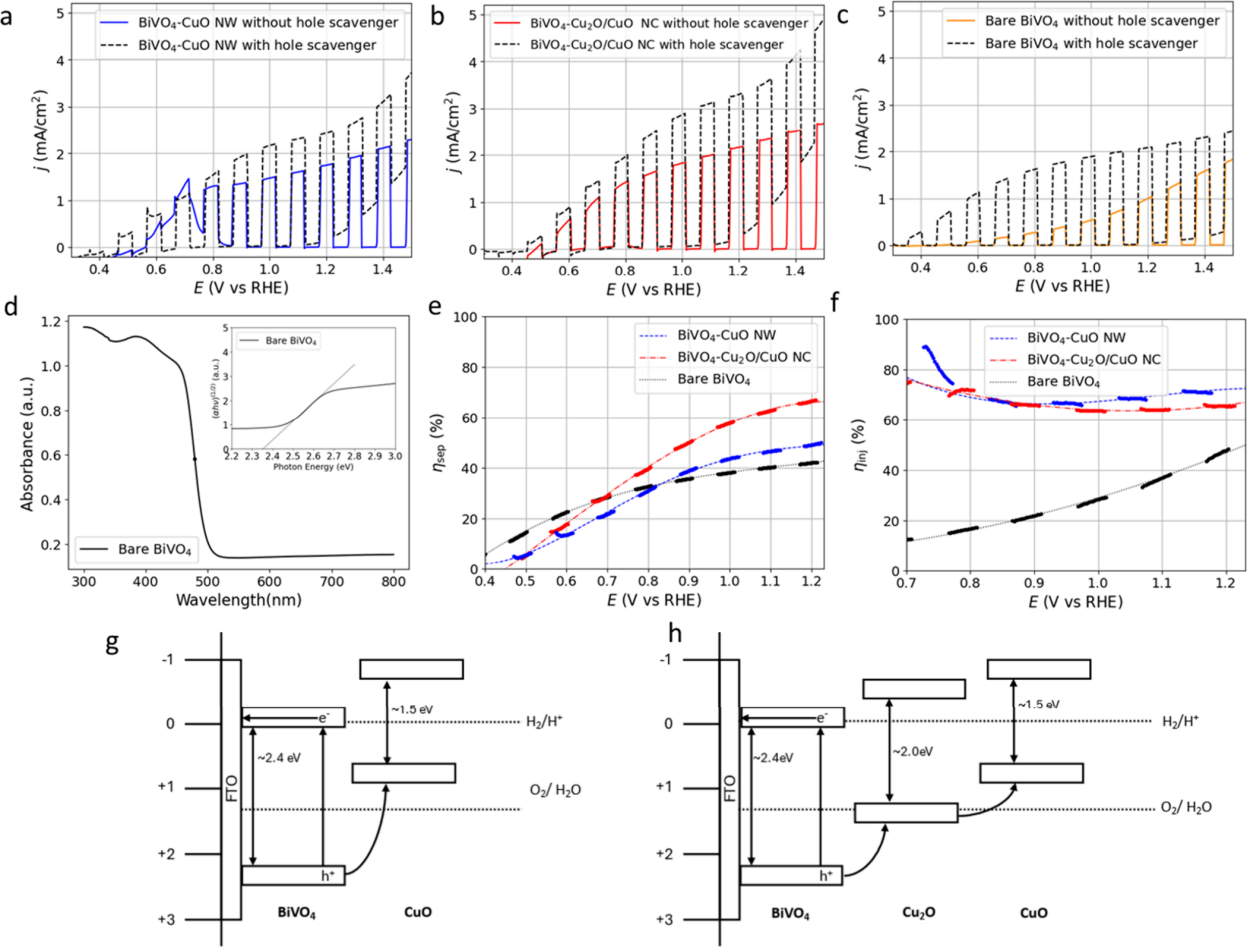
LSV curves collected with chopped AM1.5G illumination for (a) BiVO_4_–CuO NWs, (b) BiVO_4_–Cu_2_O/CuO NCs, and (c) BiVO_4_ in 1 M KCi buffer with or without 0.5 M NaSO_3_ as a hole scavenger. The scan rate is 10 mV/s. (d) UV–vis absorbance data of BiVO_4_ with inset the corresponding Tauc plot. Calculated (e) η_sep_ and (f) η_inj_, based on the data in graphs (a)–(c) and eqs 5 and 6. (g) Schematic overview of the heterojunction of BiVO_4_–CuO and (h) BiVO_4_–Cu_2_O-CuO, with band gaps based on Tauc plots for BiVO_4_ (inset of panel (d)) and Cu_2_O (Figure S20b), and a reported value for CuO elsewhere.^33^

Figure 4a compares the ratio of *j*_ph_ for two samples, one with 1 cm^2^ and the other one with a 2 cm^2^ geometrical area. For BiVO_4_–CuO NWs and BiVO_4_–Cu_2_O/CuO NCs, over 75% and 85% of *j*_ph_ are obtained when the size doubles, respectively, while a 2 cm^2^ bare BiVO_4_ sample achieves less than 65% of *j*_ph_ of a 1 cm^2^ sample (Figure S21).

**Figure 4:**
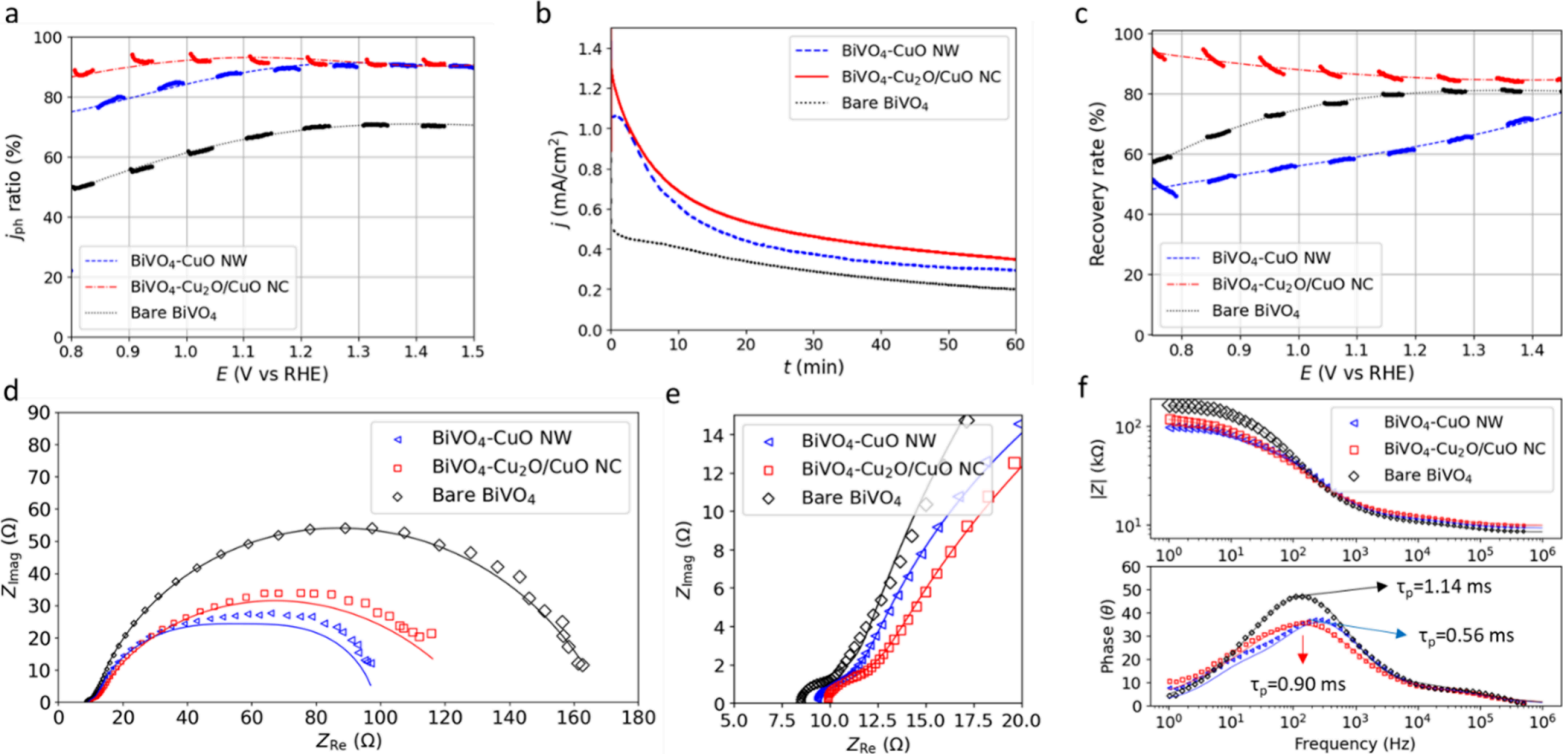
(a) Variation of current density with sample size. The figure shows the ratio of the *j*_ph_ for 1 cm^2^ vs *j*_ph_ for 2 cm^2^ of BiVO_4_–CuO NWs, BiVO_4_–Cu_2_O/CuO NCs, and bare BiVO_4_, based on LSV curves in Figure S21. (b) 1 h chronoamperometry at 0.8 V vs RHE in 1 M KCi buffer under AM1.5G irradiation. (c) Recovery rates of BiVO_4_–CuO NWs, BiVO_4_–Cu_2_O/CuO NCs, and bare BiVO_4_ samples, based on the LSV curves (Figure S22) measured in fresh electrolyte before and after the 1 h chronoamperometry as shown in panel (b). (d) Nyquist plots of BiVO_4_–CuO NWs, BiVO_4_–Cu_2_O/CuO NCs, and bare BiVO_4_. (e) Enlargement of the low impedance region of (d) and (f) Bode plots, measured at AM1.5G and 1.23 V vs RHE.

Figure 4b shows the chronoamperometry of the samples when exposed to AM1.5G-equivalent illumination for 1 h at 0.8 V vs RHE in a pH 10 1 M KCi buffer. Both combinations with copper oxide have a higher *j*_ph_ than bare BiVO_4_, with the BiVO_4_–Cu_2_O/CuO NC consistently showing the highest current density, albeit decreasing over time. After 1 h, the samples produce over a third of their initial current density, and visual damage to the samples can be observed (Figure S8). The bare BiVO_4_ sample appears thinner and shows spots without full coverage, suggesting BiVO_4_ dissolution.^10^ While the BiVO_4_–CuO NW sample shows minimal remaining CuO, its BiVO_4_ layer remains mostly intact and appears thicker than the bare BiVO_4_ sample after the chronoamperometry. The BiVO_4_–Cu_2_O/CuO NC sample retains the majority of its Cu_2_O/CuO NC layer, although spots without copper oxide are observed. Figure S22 shows LSV curves of all samples before and after the 1 h amperometry, all measured in fresh electrolyte. Based on the ratio of photocurrent measured in these graphs, we can determine the recovery rate in Figure 4c. The BiVO_4_–Cu_2_O/CuO NC sample maintains over 80% of its original *j*_ph_, suggesting that the Cu_2_O/CuO NC layer continues to function effectively. While the BiVO_4_–CuO NW sample, with limited visible CuO remaining, shows the least performance retention, it outperforms the BiVO_4_ sample after the same treatment for *E* < 1.23 V vs RHE (Figure S22e). Possibly, the CuO NW and Cu_2_O/CuO NC act as a sacrificial protection layer for the BiVO_4_ layer.

The water oxidation kinetics can be evaluated using EIS, as shown in the Nyquist plots in Figure 4d,e and the Bode Z and phase plots in Figure 4f. The Nyquist plots, measured under AM.15G-equivalent illumination and 1.23 V vs RHE, show the hole-to-water oxidation kinetics at the photoanode/electrolyte interface.^48,64^ Among the tested configurations, the BiVO_4_–CuO NW exhibits the smallest semicircle, indicating the fastest oxidation kinetics. Bare BiVO_4_ and BiVO_4_–Cu_2_O/CuO NCs can be fitted by a double parallel circuit, as seen in Figure S23a. The fitting parameters can be found in Table S3. All three Nyquist plots contain at least two semicircles, with the smallest one measured at high frequencies, independent of illumination (Figure S24). This suggests that it is unlikely to originate from defect states involved in the OER.^64^ Instead, this arc can likely be attributed to contact resistance, such as between the FTO and the BiVO_4_ layers.^48,65^ For the bigger semi arc, *R*_2_ and *C*_2_ can likely be attributed to the charge-transfer resistance *R*_t_ and the double layer capacitance *C*_dl_, respecitvely.^66^ For the BiVO_4_–CuO NW configuration, obtaining a decent fit requires either an additional parallel Randles circuit or an additional Randles circuit in series (Figures S23 and S25), likely indicating the formation of an additional surface passivation layer. Alternatively, an additional Randles circuit could be present due to additional contact resistance or the presence of defect states.^48,67^ From the Bode phase plot (Figure 4f) and eq 7, the hole lifetime is estimated to be τ_p_ = 1.14 ms for bare BiVO_4_, τ_p_ = 0.90 ms for the BiVO_4_–Cu_2_O/CuO NC combination, and τ_p_ = 0.56 ms for the BiVO_4_–CuO NW combination, again indicating that the latter has the fastest hole injection kinetics.^31,48^

The X-ray diffraction data indicate that the Cu_2_O/CuO NC consists of both CuO and Cu_2_O in the bulk. The XPS spectrum indicates that its surface consists predominantly of CuO. This is consistent with other observations as the Cu_2_O/CuO NC powder appears black, while the powder of its Cu_2_O precursor is red (Figure S26). The black powder corresponds with a composition of predominantly CuO. Additionally, structural modifications develop on the NC surfaces during calcination. In this process, CuO is formed starting from the Cu_2_O precursor (Figure S6). The spherical particles present on Cu_2_O/CuO in Figure 1f indicate that CuO is primarily formed on the surface. Finally, the Cu_2_O/CuO NCs have better catalytic properties than the Cu_2_O NCs (Figure S15a), which can be attributed to the presence of CuO on the surface. The asymmetric distortion of the surface material matters, as it has been reported that distorted CuO can enhance the water oxidation reaction. This is thought to be mediated by characteristic vibrational modes and favorable electron spin alignment to facilitate O–O bond formation.^28^ It has been reported that calcination of Cu_2_O NCs increases electrochemically active surface area and OER sites, with XPS and EDX showing surface conversion to CuO.^40,68^ DFT further suggests that Cu vacancies reduce overpotentials by introducing Cu d-orbital states near the Fermi level.^40^

The CuO NW and Cu_2_O/CuO NC catalyst perform best in a pH 10 KCi buffer, both as a standalone catalyst and integrated with BiVO_4_ (Figure 2, Figures 13 and S14). The enhanced activity may be attributed to the optimal buffering capacities of KCi near its p*K*_a_ of 10.3.^57^ Additionally, the presence of HCO_3_^–^ can facilitate proton transfer by acting as a proton acceptor.^69^

Apparently, the CuO NW or the Cu_2_O/CuO NC layer added to BiVO_4_ primarily enhanced photocurrent density compared to bare BiVO_4_ by improving charge separation and injection, rather than increasing photon absorption (Figures 3e,f). The standalone *j*_ph_ for the CuO NW and Cu_2_O/CuO NC is below 30 μA/cm^2^ (Figure S27a,b). In addition, the onset potentials for front and back illumination are comparable, while the photocurrent density is more than seven times higher when samples are illuminated on the FTO-BiVO_4_ interface compared to illumination on the BiVO_4_+copper oxide-electrolyte interface (Figure S27c,d). Finally, the UV–vis spectra (Figure S27e) of BiVO_4_–CuO NW and BiVO_4_–Cu_2_O/CuO NC show a similar reduction in absorption in the region between 450 and 500 nm to those of the pure BiVO_4_ (Figure 3d), although a significant background absorbance remains over the full measured spectrum.^63^ A small absorption shoulder at ∼570 nm in copper oxide UV–vis spectra (Figure S20a and S27e) likely originates from defect- or surface-state transitions in copper, consistent with prior reports linking similar features to copper surface chemistry and oxidation processes.^70,71^ The midpoints of both the BiVO_4_–Cu_2_O/CuO NC and BiVO_4_–CuO NW are estimated to be ∼485 nm. Finally, the IPCE, calculated with eq 8, for BiVO_4_–Cu_2_O NCs shows a similar absorption cutoff as bare BiVO_4_, both with and without a hole scavenger (Figure S28). These results confirm that photon absorption and charge carrier generation originate from the BiVO_4_ layer, with minimal contributions from the CuO NW or Cu_2_O/CuO NC layer. Thus, the performance enhancement arises from more efficient utilization of photoexcited carriers rather than increased carrier generation.

The Cu_2_O/CuO NC catalyst significantly enhanced η_sep_ observed for the BiVO_4_–Cu_2_O/CuO NC compared to bare BiVO_4_ (Figure 3e). This improvement aligns with the formation of a double Type II heterojunction structure compromising BiVO_4_–Cu_2_O-CuO layers (Figure 3h).^31^ We attribute the enhanced η_sep_ to the bulk Cu_2_O in Cu_2_O/CuO NCs, as Cu_2_O is known to function as a good hole extraction layer for BiVO_4_, and the enhanced η_inj_ to its surface CuO, supporting the dual role of the Cu_2_O/CuO NC as both a heterojunction and a cocatalyst.^17,30,38,39^ In contrast, the CuO NW layer on BiVO_4_ primarily acts as a cocatalyst as it primarily enhanced η_inj_.

The BiVO_4_–Cu_2_O/CuO NC shows improvement in η_sep_ compared to bare BiVO_4_ when *E* > 0.8 V vs RHE, with bare BiVO_4_ performing better at lower potentials. Additionally, the onset potentials of the BiVO_4_–CuO NW and BiVO_4_–Cu_2_O/CuO NC are similar, irrespective of the addition of Na_2_SO_3_ as a hole scavenger, while the onset potential of bare BiVO_4_ increased from 0.35 V vs RHE with a hole scavenger to 0.55 V vs RHE during OER. These effects might be attributed to the relatively high valence band of CuO (Figure 3g,h) close to the Fermi level (Figure S12e), compared to the lower valence band of BiVO_4_, requiring additional voltage to efficiently convert SO_3_^2–^ to SO_4_^2–^.^31,47^ At *E* > 0.8 V vs RHE, this effect might be neglected and the effect of the formation of a Type II heterojunction between BiVO_4_ and the copper oxide layer likely dominates.

The improved η_inj_ of the BiVO_4_–CuO NW and BiVO_4_–Cu_2_O/CuO NC compared to bare BiVO_4_ likely contributes to protecting BiVO_4_ against photocorrosion (Figure 4b,c, Figure S8 and S23) as less photogenerated holes are available for the oxidation of Bi­(III) due to the rapid catalysis when a copper oxide layer is added.^10^ Additionally, the rapid catalysis and improved charge separation after addition of the CuO NW or Cu_2_O/CuO NC layer on BiVO_4_ can kinetically suppress internal recombination, decreasing the dependence on the illuminated surface area compared to bare BiVO_4_ (Figure 4a, Figure S21).^72^

The BiVO_4_–Cu_2_O/CuO NC sample shows current spikes upon illumination (Figure 2d), suggesting that the rapid initial charge separation during light explosion exceeds the hole injection rate, demonstrating that higher photogenerated current densities are possible if the charge injection can be further improved.^73^

For both the BiVO_4_–CuO NW and BiVO_4_–Cu_2_O/CuO NC, at high overpotential, η_sep_ < 0.7, suggesting that further improvements are possible, such as incorporating more Type II heterojunctions to make an electron ladder for boosting efficient charge separation.^74^ Likely, additional gains for η_inj_ can be achieved using advanced cocatalysts.

The *j*_ph_ reported in this paper is comparable to that of Yang et al., who demonstrated that the combination of n-BiVO_4_ with p-Cu_*x*_O significantly enhances the oxygen evolution reaction, increasing the *j*_ph_ from 1.0 mA/cm^2^ for bare BiVO_4_ to 2.8 mA/cm^2^ for the BiVO_4_/Cu_*x*_O combination at 1.23 V vs RHE (Table S1).^31^ This enhancement is well in line with the superior catalytic properties of Cu_*x*_O compared to BiVO_4_. Those can increase η_inj_ significantly, along with a moderate improvement of η_sep_.^31^ Additionally, Meng et al. reported a BiVO_4_/CuO/TiO_2_ photoanode that achieved a relatively low *j*_ph_ of 0.48 mA/cm^2^ at 1.23 V vs RHE. Significant improvement in both η_sep_ and η_inj_ was observed compared to bare BiVO_4_.^37^ Furthermore, Murugan and Pandikumar achieved a *j*_ph_ of 2.05 mA/cm^2^ at 1.23 V vs RHE with their BiVO_4_–CuO combination, which was again attributed to the improved separation of electron–hole pairs and enhanced hole transfer at the electrolyte interface.^63^ Finally, Li et al. reported a BiVO_4_/Cu_2_O/Co-Pi photoanode reaching 2.22 mA/cm^2^.^75^ Well in line with earlier results, we find for two different routes, starting from Cu­(OH)_2_ or Cu_2_O, that the BiVO_4_–CuO NW and BiVO_4_–Cu_2_O/CuO NC can be prepared and can show comparable *j*_ph_ (Table S1).^31,63^ In particular, the CuO NW derived from Cu­(OH)_2_ moderately improves η_sep_, while the addition of Cu_2_O/CuO NCs, derived from Cu_2_O, significantly increases η_sep_. These differences indicate that the copper oxide morphology affects the photoelectrochemical performance and point to a significant contribution of Cu_2_O in Cu_2_O/CuO to the increase in η_sep_.

The morphologies with associated lattice deformations that appear after calcination on the CuO NW and Cu_2_O/CuO NC surfaces (Figure 1) most likely facilitate the relatively high η_inj_. It was proposed that the asymmetry of curvature in mesocrystals of cupric oxide derived from Cu­(OH)_2_ makes nonadiabatic conversion by adiabatic passage possible, related to the quasi-1D chainlike CuO_*x*_ structure.^34,51^ The CuO NW and Cu_2_O/CuO NC samples exhibit a consistent Tafel slope at *j* > 2 mA/cm^2^, in contrast with their precursors, indicating rapid catalysis (Figure S15a). The high Tafel slopes compared to copper oxide nanoparticles on metal substrates can be attributed to the use of FTO as a background substrate, as OER catalysts on transparent conductive layers are known to display low catalytic rates.^28,76–78^

Variation in the catalyst loading on BiVO_4_ directly affects the photocurrent response. For example, in Figure S29, the catalyst loading was reduced from the standard 0.6 mg/cm^2^ to 0.3 mg/cm^2^, resulting in a diminished photocurrent for both BiVO_4_–CuO NW and BiVO_4_–Cu_2_O/CuO NC photoanodes.

Copper oxides are known to be unstable under OER conditions (Figure 4b,c, Figure S8).^27^ Adding an ultrathin protection layer on top of metal oxide layers can prevent degradation.^79^ For example, an ultrathin ALD TiO_2_ layer is known to significantly improve the stability of photoelectrodes and can effectively eliminate defect states in the BiVO_4_–CuO combination while facilitating hole tunneling into the electrolyte, thereby making CuO a more promising material for future applications.^37,80^

## Conclusions

Two distinct copper oxide nanostructures were successfully drop-cast on BiVO_4_, both significantly enhancing the photogenerated current densities. The BiVO_4_–CuO NW combination primarily acted as a cocatalyst, with slight improvements in η_sep_, but most in the water oxidation kinetics. The BiVO_4_–Cu_2_O/CuO NC combination functioned as both a Type II heterojunction and a cocatalyst, increasing η_sep_ as well as η_inj_, achieving the most improvement in the photogenerated current density. The increase in η_inj_ is attributed to the small spherical CuO particles observed on both the Cu_2_O/CuO NC and CuO NW structures, while the η_sep_ is attributed to bulk Cu_2_O in the Cu_2_O/CuO NCs. Additionally, selecting the buffer type, pH, and molarity is essential to obtaining high photogenerated current densities, with a pH 10 1 M KCi buffer being the most optimal for the BiVO_4_–Cu_2_O/CuO NC photoanode.

## Supplementary Material



## Notes

Additional tables, photographs of samples and setup, SEM images, XRD spectra, XPS spectra, LSV measurements, UV–vis measurements, EIS measurements, and IPCE measurements (PDF)

## ABBREVIATIONS

ABPE: Applied Bias Photon-to-Current Efficiency
ALD: Atomic Layer Depositon
BiVO_4_: Bismuth Vanadate
CE: Counter Electrode
Cu_(*x*)_O: Copper Oxide
CuO: Cupric Oxide
Cu_2_O: Cuprous oxide
CV: Cyclic Voltammetry
EIS: Electronic Impedance Spectroscopy
FTO: Fluoride-Doped Tin Oxide
KBi: Potassium Borate
KCi: Potassium Carbonate
KPi: Potassium Borate
IPCE: Incident Photon to Current Efficiency
LSV: Linear Sweep Voltammetry
MQ: Milli-Q
NC: Nanocubes
NW: Nanowires
PEC: Photoelectrochemical
RHE: Reversible Hydrogen Electrode
SEM: Scanning Electron Microscopy
SEM-EDX: Scanning Electron Microscopy with Energy-Dispersive X-ray Spectroscopy
WE: Working Electrode
XRD: X-ray Diffraction
