# Supplementary material for: BiVO4–Cu2O/CuO Nanocubes with High Charge Injection and Charge Separation Rates for Enhanced Photoelectrochemical Water Oxidation
Source: ACS Appl Energy Mater. 2025 Oct 30;8(21):15746–57. doi: 10.1021/acsaem.5c02177 (PMC12606557; doi:10.1021/acsaem.5c02177)
Supplement: Supplementary file 1 [file ae5c02177_si_001.pdf]

# Supporting Information

# $BiVO_4$-$Cu_2O$/CuO Nanocubes with high charge injection and charge separation rates for enhanced photoelectrochemical water oxidation.

*Suzanne M.E. Assen[a], Willemijn H. Boeije[a], Pieter de Haij[a], Camilo A. Mesa[b], Ana Gutiérrez-Blanco[b], Laura Montañés[b], Sixto Giménez*[b],Huub J.M. de Groot*[a]*

AUTHOR ADDRESS

[a] Leiden Institute of Chemistry, Leiden University, Einsteinweg 55, 2300 RA, Leiden, The Netherlands

[b] Institute of Advanced Materials (INAM), Universitat Jaume I, Avenida de Vicent Sos Baynat, s/n,12006 Castellón de la Plana, Spain

*ssnmr@chem.leidenuniv.nl

* sjulia@uji.es

Table S1: Overview of $BiVO_4/Cu_xO$ structures for photoelectrochemical water oxidation

| Photoanode Structure | Electrolyte | Current density at 0.85 V vs RHE ($mA/cm^2$) | Current density at 1.23 V vs RHE ($mA/cm^2$) | Source |
|---|---|---|---|---|
| $BiVO_4/Cu_xO$ | 0.2 M NaPi buffer | ~1.55 | ~2.8 | Yang et al.[1] |
| $BiVO_4/CuO/TiO_2$ | 0.5 M $Na_2SO_4$ | ~0.3 | 0.48 | Meng et al.[2] |
| $BiVO_4/CuO$ | 0.1 M KPi buffer | ~0.5 | 2.05 | Murugan & Pandikumar[3] |
| $BiVO_4/Cu_2O$/Co-Pi | 0.1 M KPi buffer | ~1.6 | 2.22 | Li et al.[4] |
| $BiVO_4/Cu_2O$/Co-Pi | 0.1 M KPi buffer | ~1.5 | 1.97 | Yang et al.[5] |
| $BiVO_4$/rGO/$Cu_2O$ | 0.1 M KPi buffer | ~0.6 | ~1.4 | Bai et al.[6] |
| $BiVO_4/Cu_2O$ | 0.1 M KPi Buffer | ~1.0 | 1.72 | Bai et al.[7] |
| **Bare $BiVO_4$** | **1 M KCi buffer** | **0.68** | **1.39** | **This work** |
| **$BiVO_4$-CuO NW** | **1 M KCi buffer** | **1.39** | **2.10** | **This work** |
| **$BiVO_4$-$Cu_2O$/CuO NC** | **1 M KCi buffer** | **1.58** | **2.25** | **This work** |

Table S2: XRD data from Figure 1g (main text) and Figures S9-S11. Peak location and FWHM are determined by hand in 2θ units. For use in the Scherrer equation, and peak location is converted into θ and FWHM to radian units.

| Sample | Figure | Reflection location (2θ) | FWHM (2θ) | Size (nm) |
|---|---|---|---|---|
| $BiVO_4$ on FTO | 1g (main text) | 28.9±0.05 | 0.20±0.05 | 41±11 |
| $BiVO_4$ on FTO | 1g (main text) | 30.55±0.05 | 0.14±0.03 | 59±12 |
| CuO NW powder | S11 | 35.2±0.2 | 1.1±0.2 | 7.6±1.4 |
| CuO NW powder | S11 | 38.5±0.2 | 1.15±0.2 | 7.3±1.3 |
| CuO NW on FTO | S9 | 35.4±0.3 | 0.7±0.2 | 12±3 |
| CuO NW on FTO | S9 | 39.1±0.3 | 0.8±0.2 | 11±3 |
| $Cu(OH)_2$ NW on FTO | S9 | 23.65±0.2 | 1.0±0.2 | 8.1±1.6 |
| $Cu(OH)_2$ NW powder | S11 | 33.9±0.2 | 1.1±0.2 | 7.6±1.4 |
| $Cu_2O$ NC powder | S11 | 36.25±0.05 | 0.25±0.02 | 33±3 |
| $Cu_2O$ NC on FTO | S10 | 36.55±0.05 | 0.20±0.04 | 42±8 |
| $Cu_2O$ of $Cu_2O$/CuO NC powder | S11 | 36.1±0.1 | 0.25±0.02 | 33±3 |
| CuO of $Cu_2O$/CuO NC powder | S11 | 35.3±0.3 | 0.6±0.2 | 14±5 |
| CuO of $Cu_2O$/CuO NC powder | S11 | 38.5±0.3 | 0.8±0.3 | 11±4 |
| $Cu_2O$ of $Cu_2O$/CuO on FTO | S10 | 36.55±0.05 | 0.20±0.02 | 42±4 |
| CuO of $Cu_2O$/CuO NC on FTO | S10 | 35.6±0.1 | 0.8±0.2 | 10±2 |
| CuO of $Cu_2O$/CuO NC on FTO | S10 | 38.8±0.2 | 0.9±0.4 | 9.4±5 |

Table S3: EIS fitted parameters. The fits used in Figure 4d-f are made bold. $R^2$ is calculated based on the combination of the absolute value of Z and the phase plots.

| Structure | Model | Rs(Ω) | R1(Ω) | R2(Ω) | R3(Ω) | Q1 ($F \cdot s^{n1-1}$) | Q2 ($F \cdot s^{n2-1}$) | Q3 ($F \cdot s^{n2-1}$) | n1 | n2 | n3 | $R^2$ |
|---|---|---|---|---|---|---|---|---|---|---|---|---|
| **Bare $BiVO_4$** | **S23a** | **8.3 ± 0.6** | **156 ± 1** | **5.7 ± 0.7** | **-** | **3.5e-5 ± 9e-6** | **1.5e-4 ± 2e-5** | **-** | **0.90 ± 0.02** | **0.65 ± 0.02** | **-** | **0.9996** |
| **$BiVO_4$-$Cu_2O$/CuO NC** | **S23a** | **9.6± 0.4** | **113 ± 4** | **8 ± 3** | - | **4e-5 ± 3e-5** | **4.8e-4 ± 3e-5** | - | **0.85 ± 0.06** | **0.55 ± 0.03** | - | **0.9999** |
| $BiVO_4$/CuO NW fit 1 (Figure S25a) | S23a | 9.2 ± 0.1 | 56±1 | 3.2 ± 0.4 | - | 8e-5 ± 3e-5 | 2e-5 ± 1e-5 | - | 0.82 ± 0.01 | 0.80 ± 0.05 | - | 0.887 |
| $BiVO_4$/CuO NW fit 2 (Figure S25b) | S23a | 10.0 ± 0.1 | 18 ± 3 | 72 ± 3 | - | 7.1e-4 ± 9e-5 | 1.6e-4 ± 2e-5 | - | 1.00 ± 0.06 | 0.74 ± 0.01 | - | 0.9998 |
| **$BiVO_4$/CuO NW Fit 4(Figure 25c)** | **S23b** | **9.1 ± 0.2** | **52± 7** | **4 ± 2** | **33± 9** | **5e-5 ± 4e-5** | **1e-4 ± 9e-5** | **7e-4 ± 1e-4** | **0.86 ± 0.09** | **0.68 ± 0.12** | **0.92 ± 0.05** | **0.9999** |
| $BiVO_4$/CuO NW Fit 3 (Figure 25d) | S23c | 9.0 ± 0.2 | 3.2 ± 1.2 | 49 ± 8 | 44 ± 9 | 2.5e-5 ± 5e-6 | 6e-5 ± 4e-5 | 1.2e-3 ± 2e-4 | 0.79 ± 0.19 | 0.85 ± 0.07 | 0.66 ± 0.07 | 0.9992 |

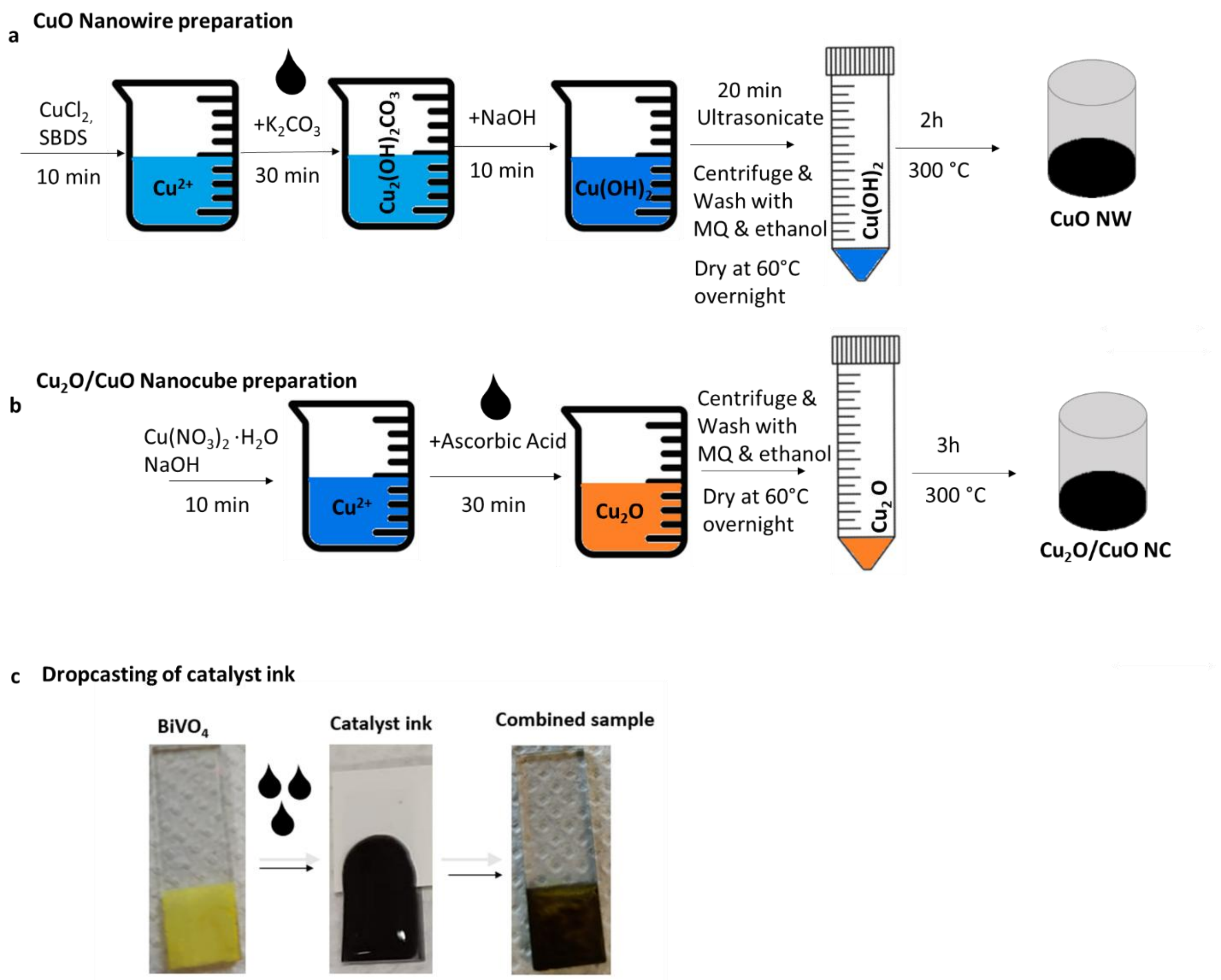


Figure S1: Preparation steps to make a) CuO NW and b) $Cu_2O$/CuO NC. c) Subsequent drop-casting of the prepared powder on top of $BiVO_4$.

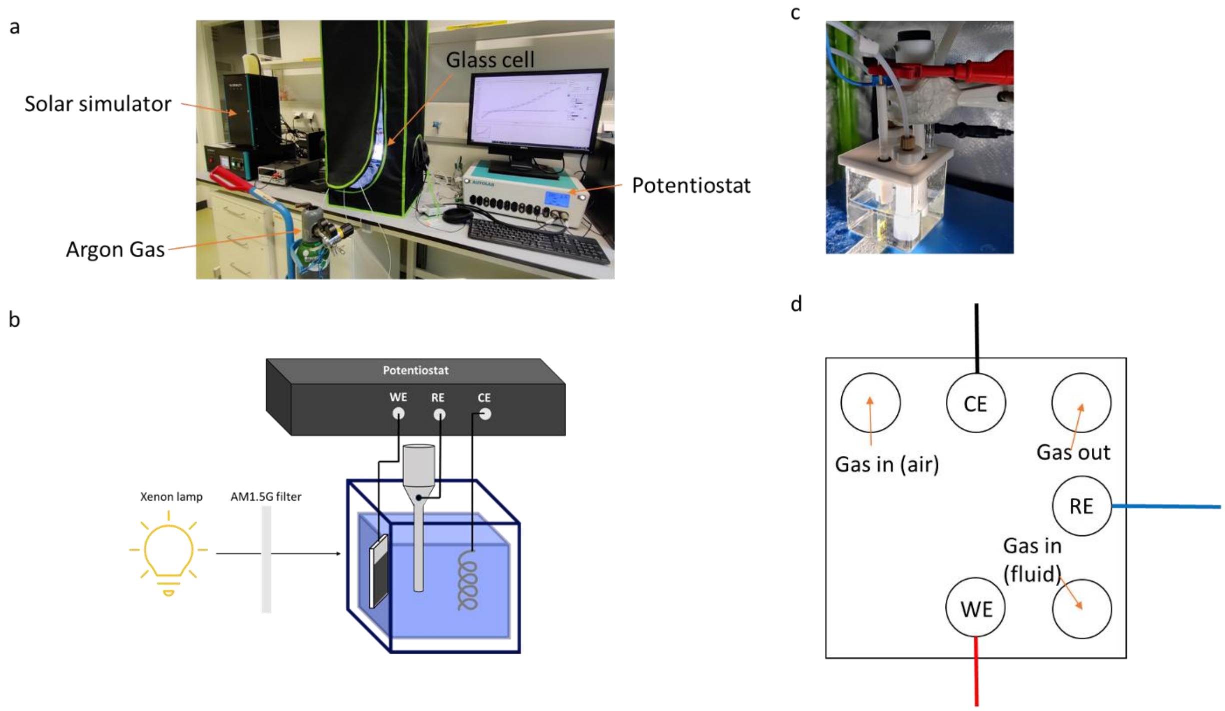


Figure S2: The photoelectrochemical set-up. a) Photographic and b) schematic overview of the entire photoelectrochemical setup under operation. WE is working electrode RE is reference electrode, and CE is counter electrode. In the schematic overview, the gas in and outlets are not depicted. c) Close up of the quartz glass cell used under operation. d) Schematic overview of the top of the glass cell, with gas inlets and outlet. The outlet in the right bottom does insert in the fluid and is only used before electrochemical experiments. The outlet on the upper left inserts above the normal fluid level and gas is let in during electrochemical measurements.

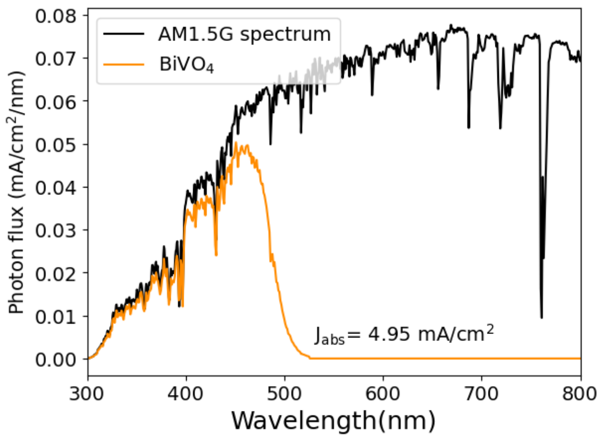


Figure S3: The UV-Vis absorbance mapped on the AM1.5G spectrum to calculate the $j_{\mathrm{abs}}$.

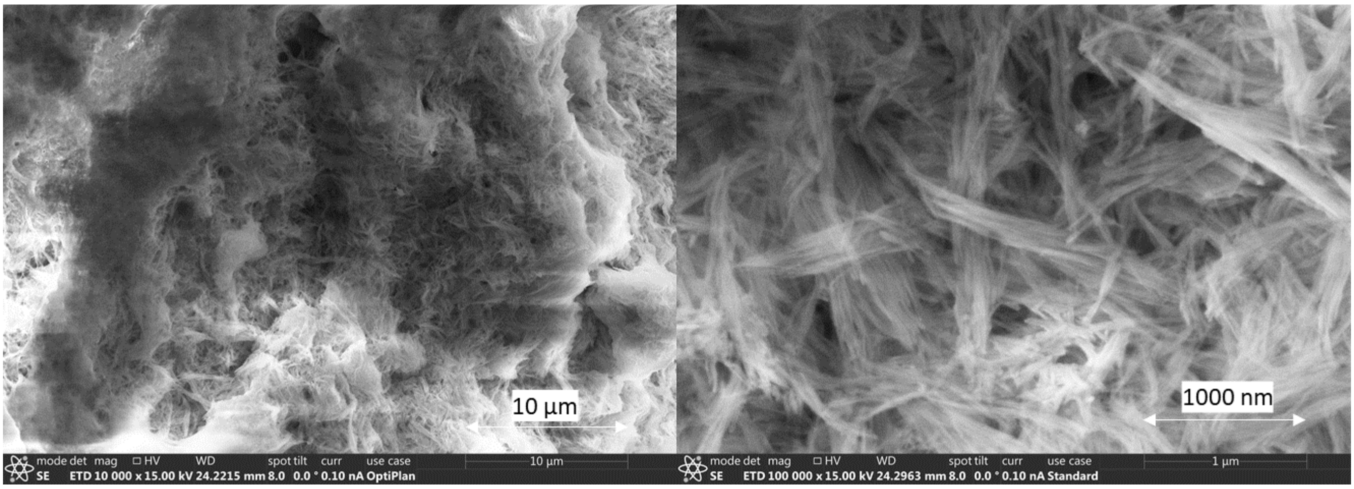


Figure S4: SEM images of the $Cu(OH)_2$ NWs precursor on FTO. The $Cu(OH)_2$ NWs are prepared as CuO NW described in the main text without the final calcination step. They were drop-casted on clean FTO.

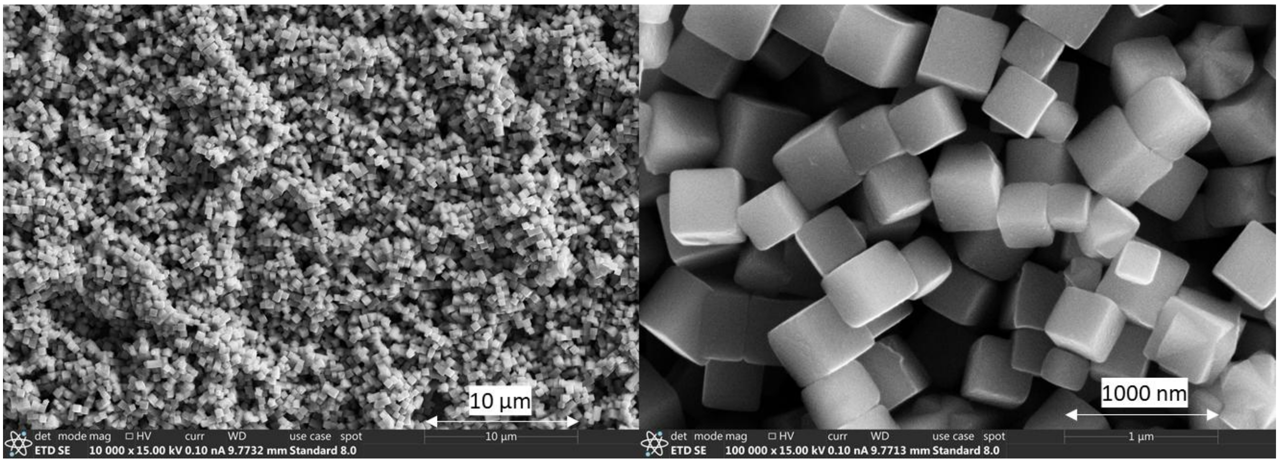


Figure S5: SEM images of the $Cu_2O$ NC precursor on FTO. The $Cu_2O$ NC was prepared as the $Cu_2O/CuO$ NC described in the main text without any calcination. They were drop-casted on clean FTO.

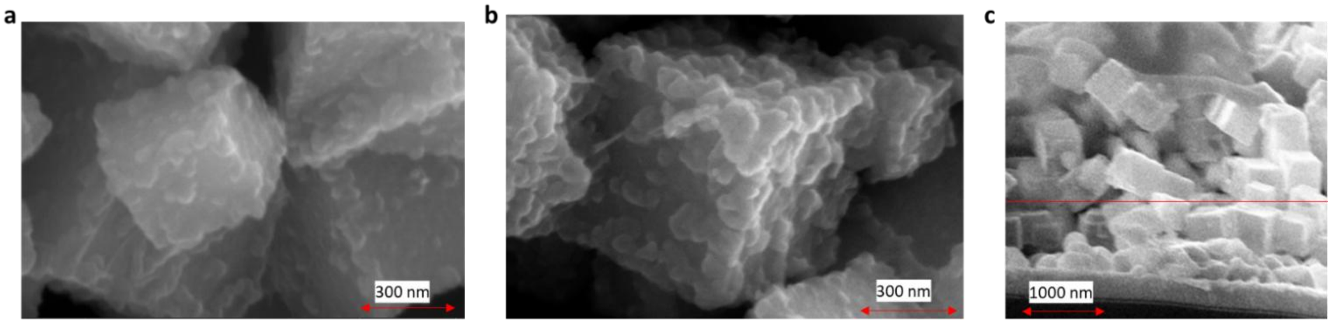


Figure S6: a) and b) Zoomed-in SEM image of $Cu_2O$/CuO NC. c) Sideways image of $BiVO_4$-$Cu_2O$/CuO NC. The $BiVO_4$ lays on top of an FTO layer, with $Cu_2O$/CuO NC on top of the $BiVO_4$

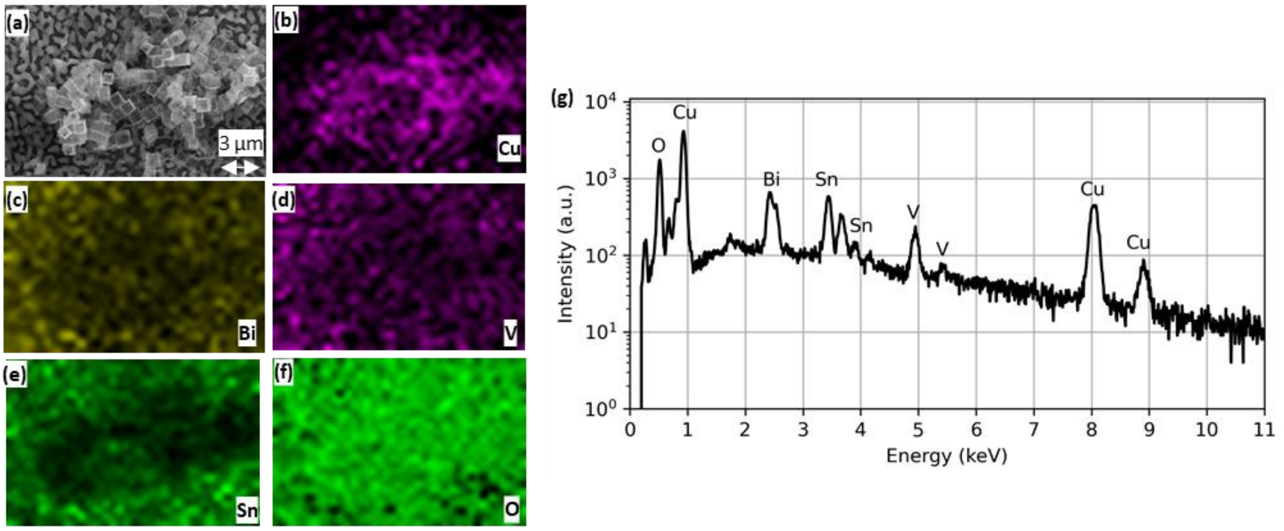


Figure S7: EDX spectrum of $BiVO_4$-$Cu_2O$/CuO NC near the edge of the sample, with (a) the SEM image and (b-f) the spectra associated with Cu, Bi, V, Sn and O respectively and (g) the associated energy.

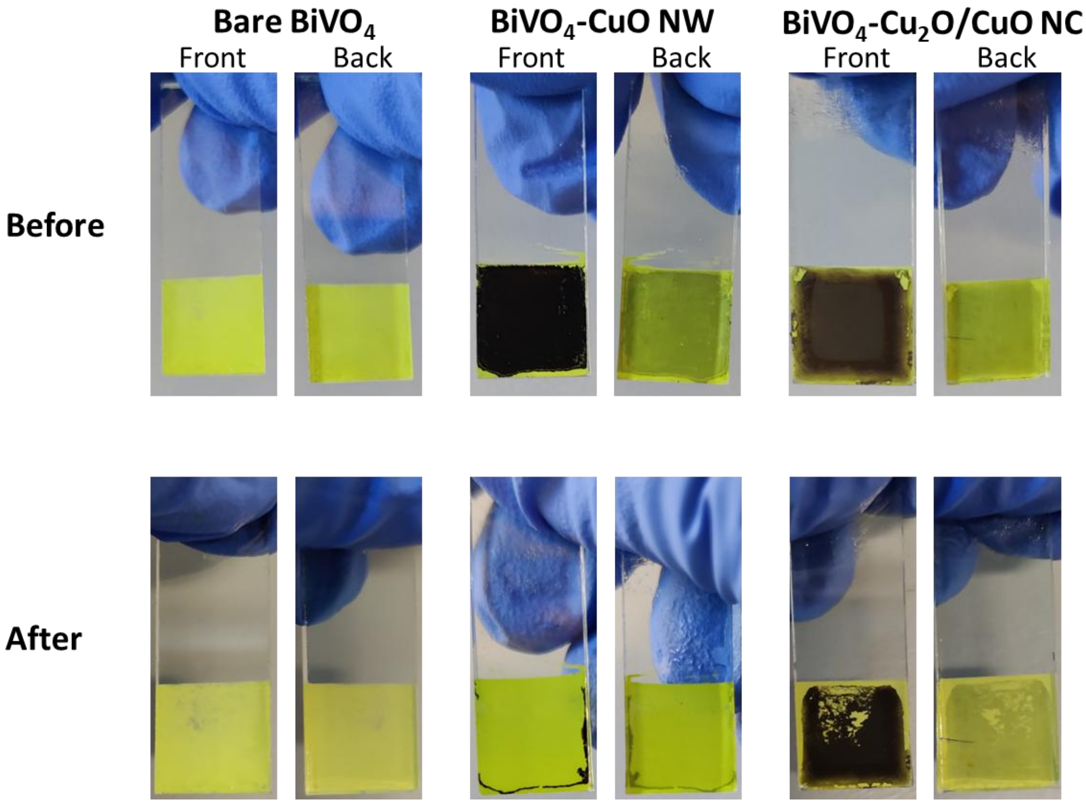

Figure S8: Photographs of the back and front of the bare $BiVO_4$, $BiVO_4$-CuO NW and $BiVO_4$-$Cu_2O$/CuO NC samples, before and after one hour amperometry at 0.8V vs RHE (Figure 4b), illuminated using 1000 W/$m^2$.

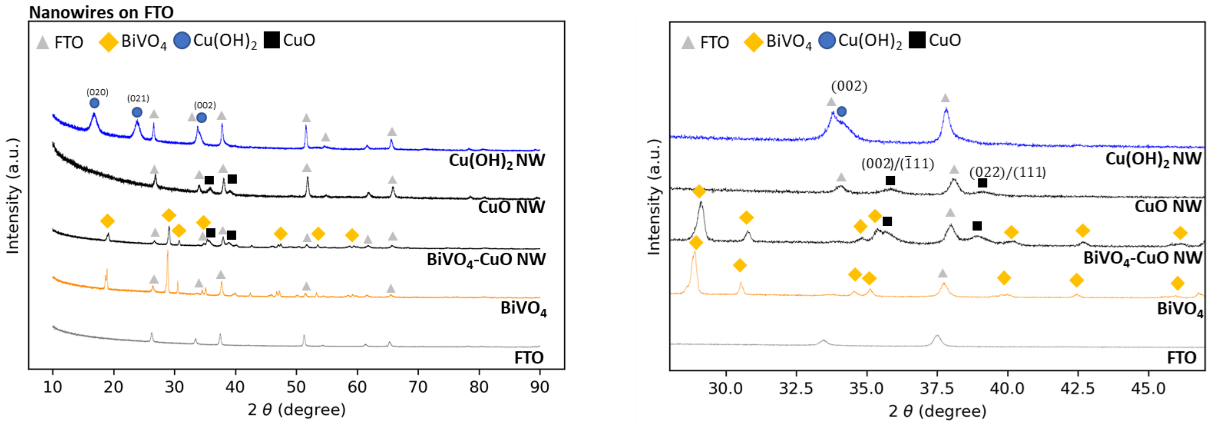


Figure S9: XRD spectra of the $BiVO_4$-CuO NW combination on FTO with its parent materials of bare FTO, bare $BiVO_4$ on FTO, bare CuO NW on FTO and the intermediate material $Cu(OH)_2$ on FTO. The samples are compared and matched against ICSD reference spectra, with collection codes 154960, 33243, 68459, 52043 and 69757 for $SnO_2$, $BiVO_4$, $Cu(OH)_2$, $Cu_2O$, and CuO respectively.

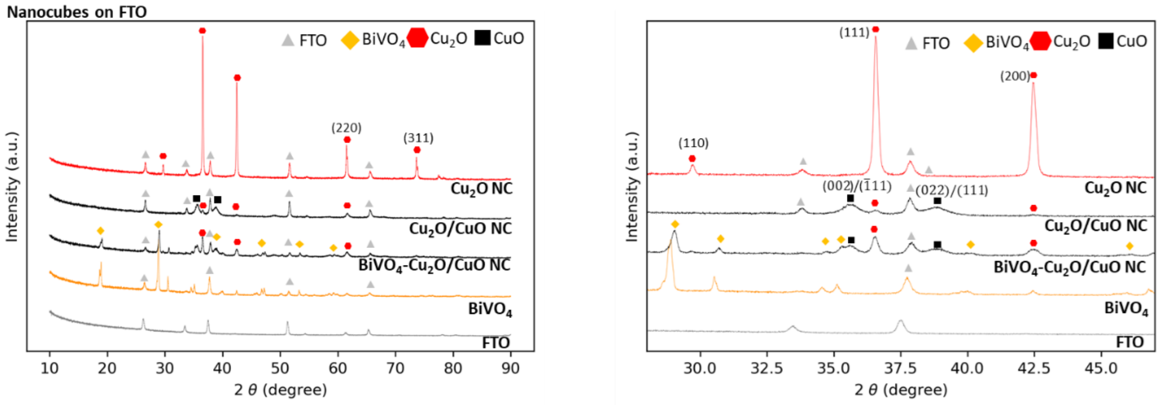


Figure S10: XRD spectra of the $BiVO_4$-$Cu_2O$/CuO NC combination on FTO with its parent materials of bare FTO, bare $BiVO_4$ on FTO, bare $Cu_2O$/CuO NC on FTO and the intermediate material $Cu_2O$ NC on FTO. The samples are compared to ICSD reference spectra for $SnO_2$, $BiVO_4$, $Cu_2O$, and CuO with collection codes 154960, 33243, 52043 and 69757, respectively.

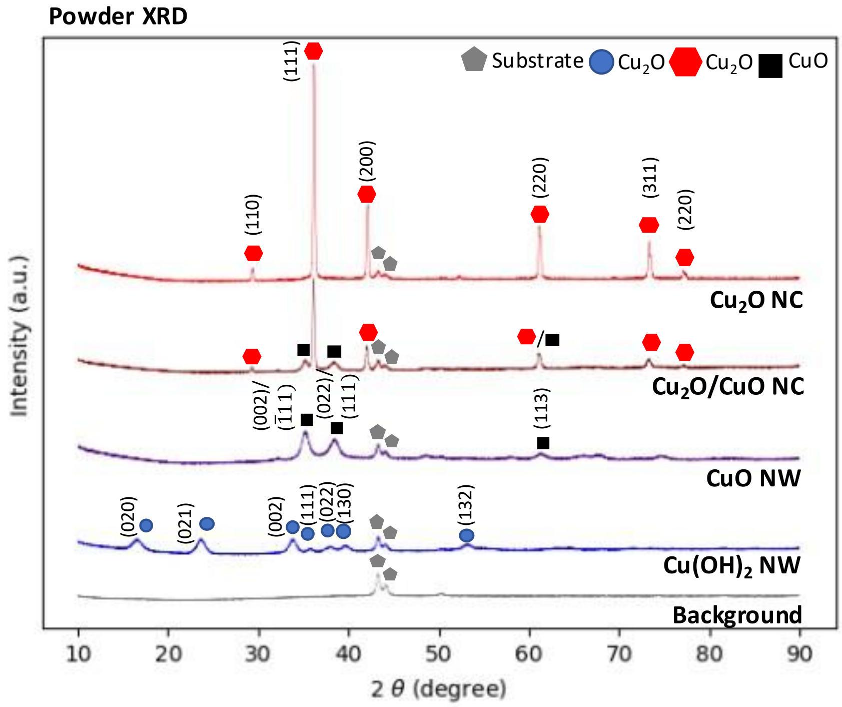


Figure S11: The XRD spectra of the CuO NW and $Cu_2O$/CuO NC powders and their precursors. The peaks are matched against ICSD reference spectra, with collection codes 68459, 52043 and 69757 for $Cu(OH)_2$, $Cu_2O$, and CuO respectively.

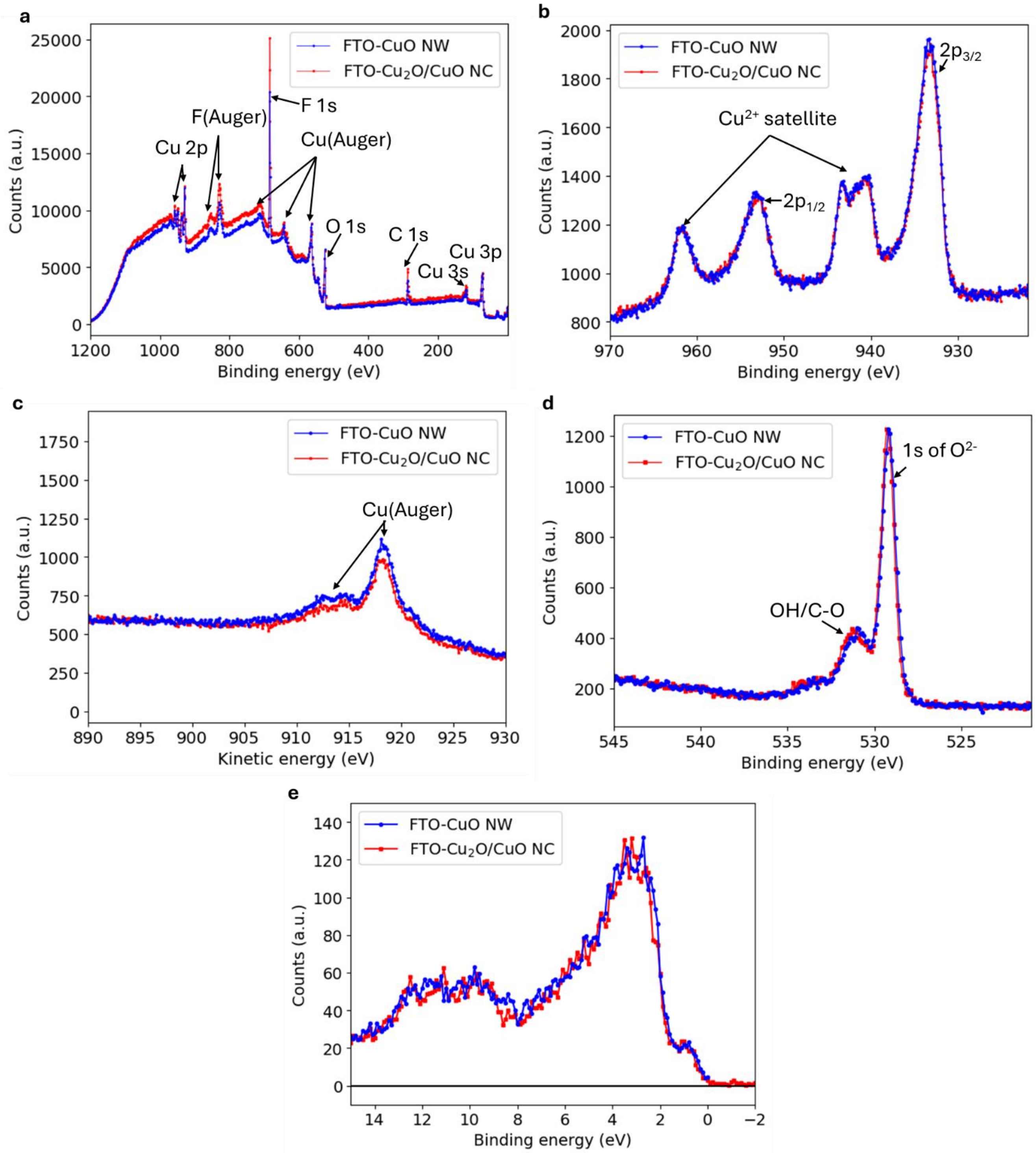


Figure S12: XPS spectra of FTO–CuO NW and FTO–$Cu_2O$/CuO NC samples. (a) Full survey spectrum showing signals from Cu 2p, F 1s, O 1s, C 1s, Cu 3s, and Cu 3p.[8] (b) High-resolution Cu 2p region with Cu $2p_3/_2$ and Cu $2p_1/_2$ peaks, along with intense shake-up satellites characteristic of $Cu^{2+}$.[9,10] (c) Cu LMM Auger spectrum. (d) O 1s region, with the peak at 529 eV attributed to $O^{2-}$ and at 531 eV to either OH bond or C-O bonds[10]. (e) Valence band spectrum.

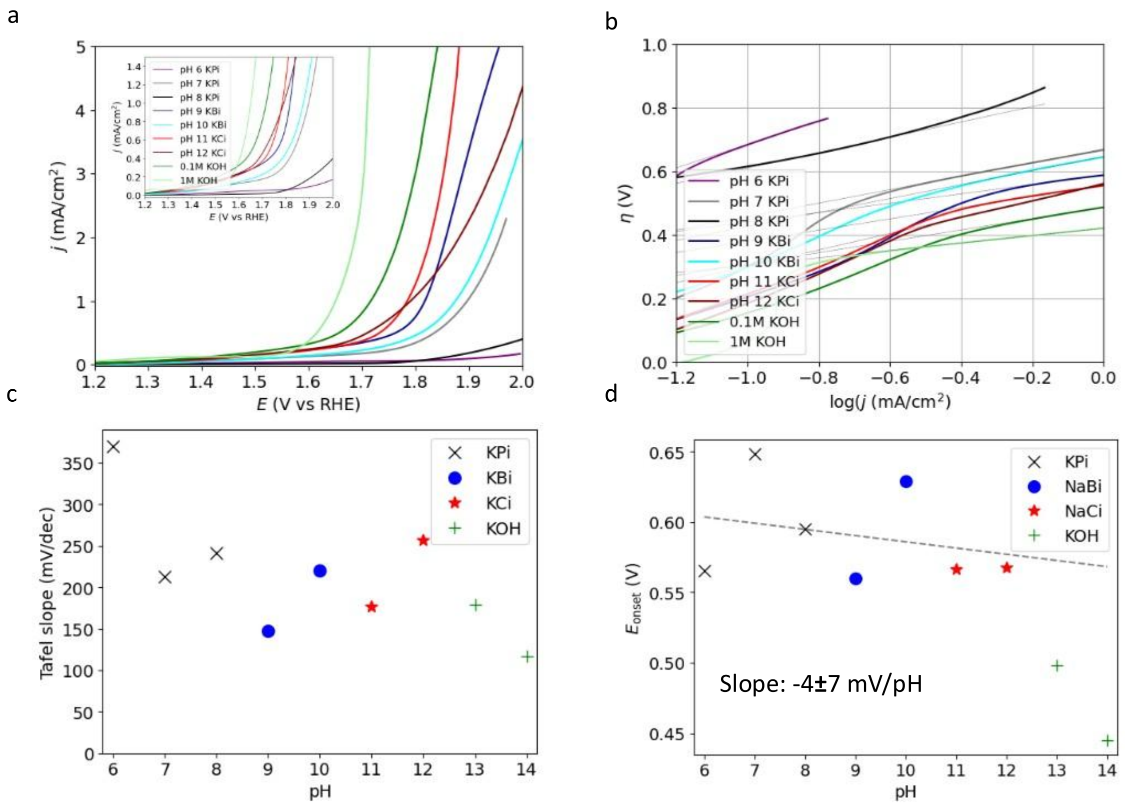


Figure S13: CuO NW on FTO in KPi, KBi, KCi and KOH buffers with different pH. a) shows the LSV curves of the samples, with the inset showing a zoomed in plot at low current densities. The dashed line indicates the onset potential. b) shows the fitted Tafel slopes. c) shows the Tafel slope vs pH and d) shows the onset potential as determined with the dashed lines in a).

The LSV curves were measured in a custom made 3-electrode setup with a 3 M KCl Ag/AgCl electrode as the reference electrode. The scan speed was 10 mV/s. The potential $V_{\mathrm{RHE}} = V_{\mathrm{Ag/AgCl}} + 0.210 + 0.591 * \mathrm{pH} - j \cdot R$. To obtain the most accurate values, the Tafel plot was fitted on its most linear part, using at least $j > 0.1$ mA/cm$^2$.[11]

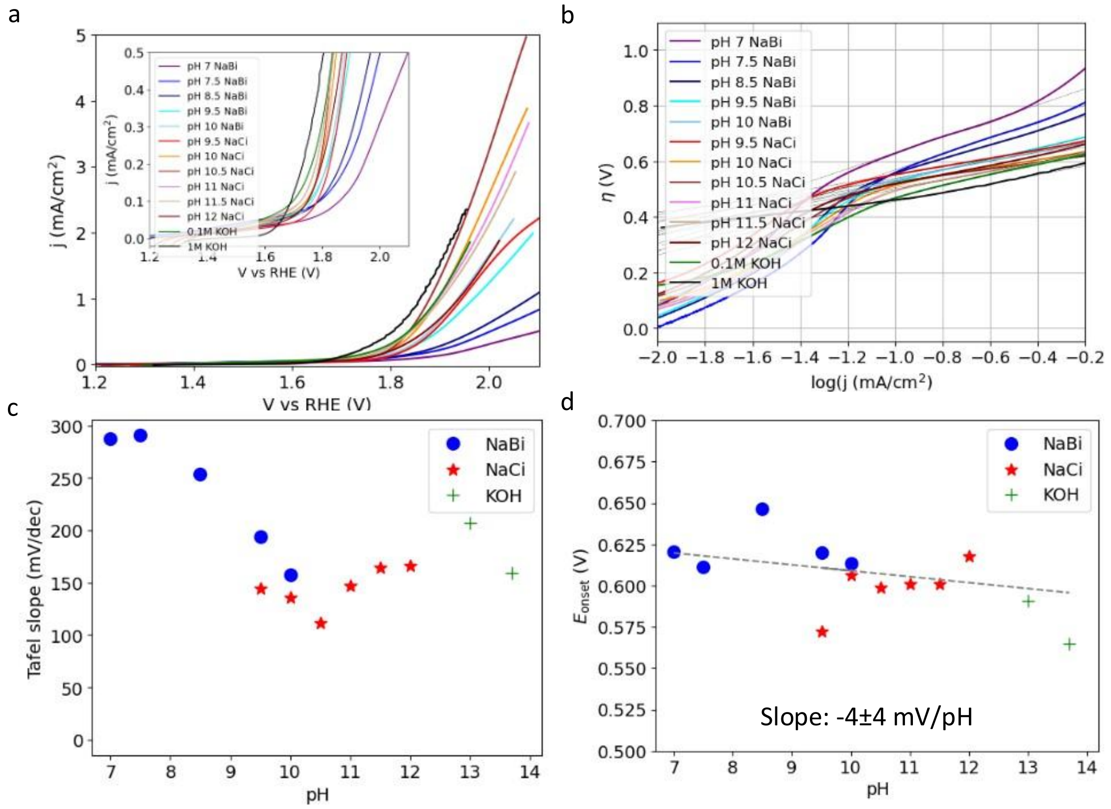


Figure S14: $Cu_2O/CuO$ NC on FTO in NaBi, NaCi and KOH buffers with different pH. a) shows the LSV curves of the samples, with the inset showing a zoomed in plot at low current densities. The dashed line indicates the onset potential. b) shows the fitted Tafel slopes. c) shows the Tafel slope vs pH and d) shows the onset potential as determined with the dashed lines in a).

The LSV curves were measured in a custom made 3-electrode setup with a 3 M KCl Ag/AgCl electrode as the reference electrode. The scan speed was 50 mV/s. The potential $V_{\mathrm{RHE}} = V_{\mathrm{Ag/AgCl}} + 0.210 + 0.591 * \mathrm{pH} - j \cdot R$. To obtain the most accurate values, the Tafel plot was fitted on its most linear part, using at least $j > 0.1$ mA/cm². [11]

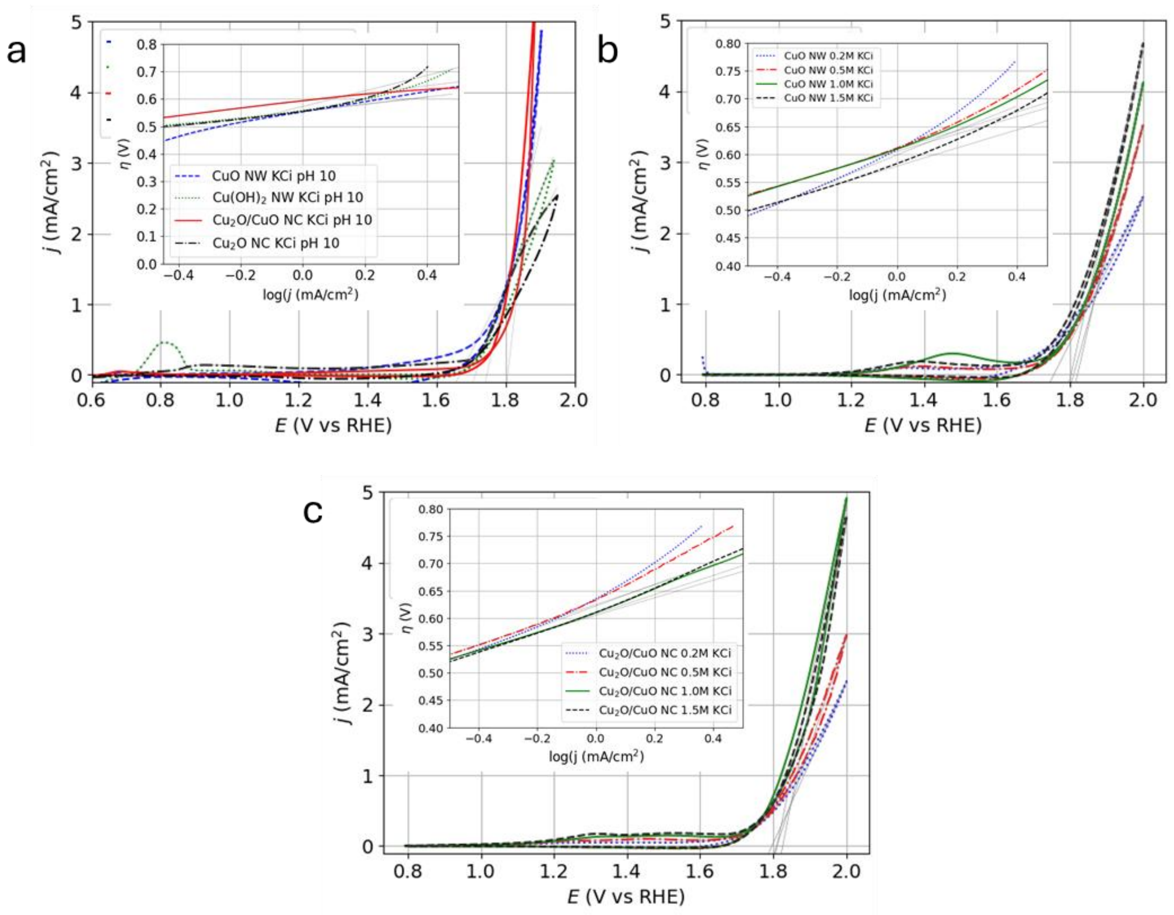


Figure S15: CVs of a) CuO NW, $Cu(OH)_2$ NW, $Cu_2O$/CuO NC and $Cu_2O$ NC at 1 M KCi pH 10. b) CuO NW and c) $Cu_2O$/CuO NC at different molarity concentrations for a pH 10 KCi buffer. The scan rate is 10 mV/s and resistance is not corrected.

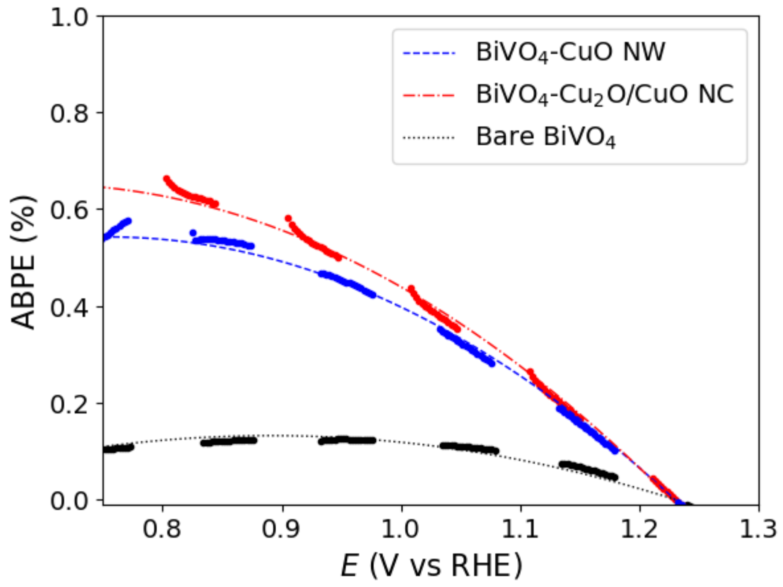


Figure S16: Applied Bias Photon-to-current Efficiency (ABPE), based on the chopped illuminated LSVs in Figure 2d for $BiVO_4$-CuO NW, $BiVO_4$-$Cu_2O$/CuO NC and $BiVO_4$, respectively, calculated with eq 4.

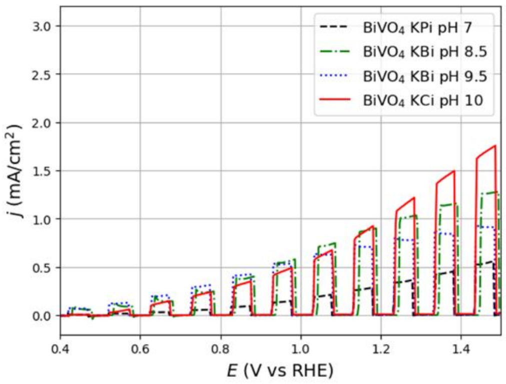

Figure S17: LSV curves under chopped light illumination of a single sample of bare $BiVO_4$ in different buffers with increasing pH. The scan rate was 10 mV/s.

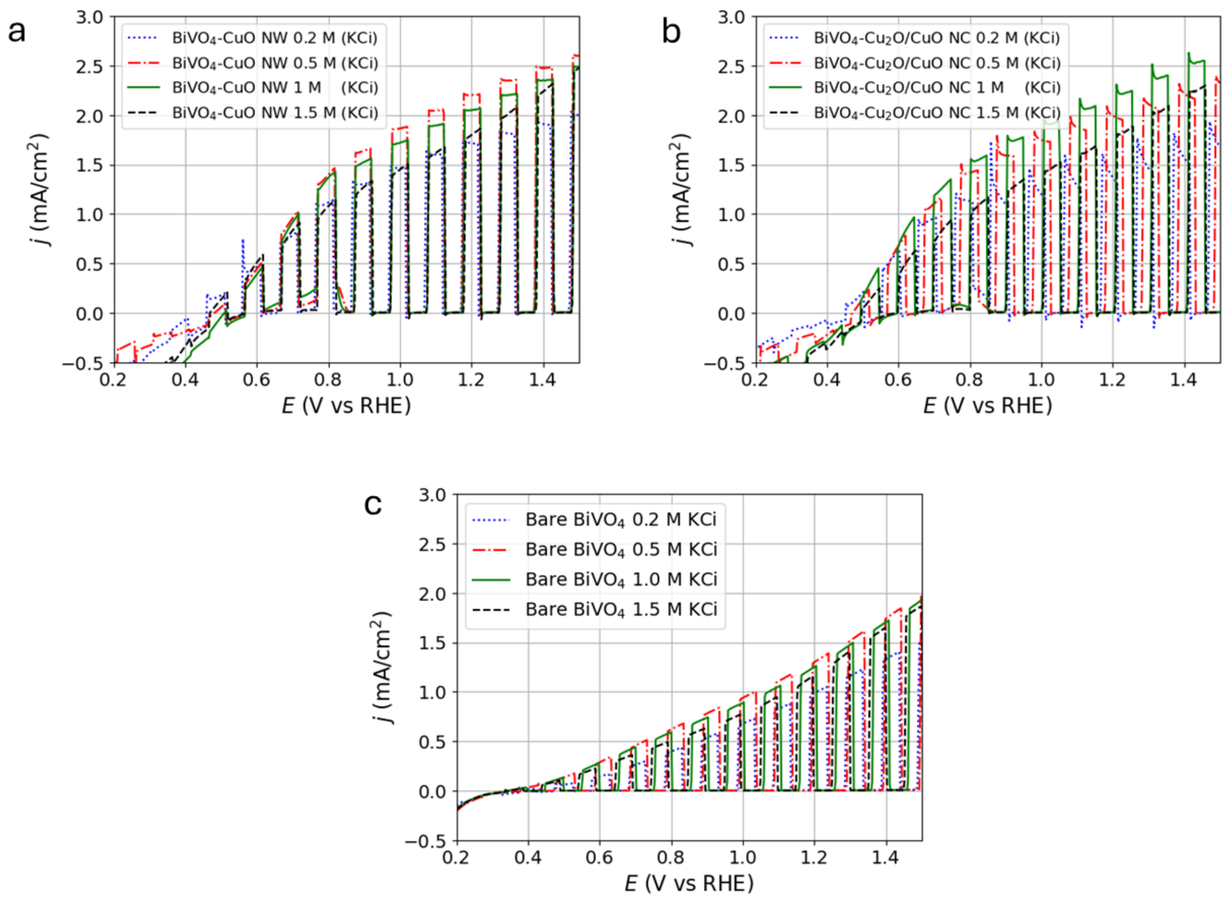


Figure S18: a) $BiVO_4$-CuO NW, b) $BiVO_4$-$Cu_2O$/CuO NC, and c) Bare $BiVO_4$ in different concentrations of a KCi pH 10 buffer at chopped AM1.5G. The scan rate was 10 mV/s.

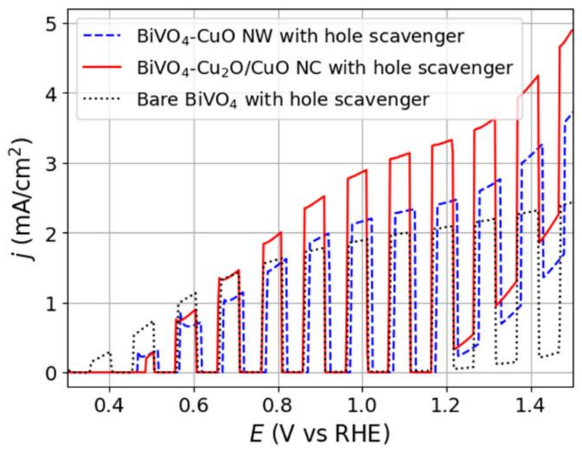


Figure S19: Measured chopped LSV curves of $BiVO_4$-CuO NW, $BiVO_4$-$Cu_2O$/CuO NC and bare $BiVO_4$ in 1 M KCi buffer with 0.5 M $NaSO_3$ as a hole scavenger under chopped AM1.5G illumination in one graph. The scan rate was 10 mV/s.

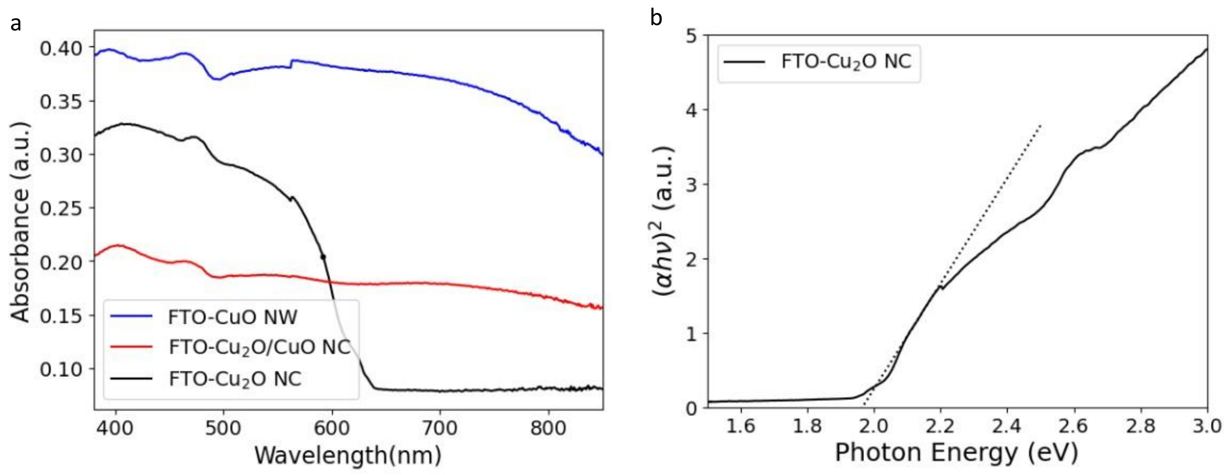


Figure S20: (a) UV-Vis of CuO NW, $Cu_2O$/CuO NC and $Cu_2O$ NC on FTO with the midpoint of $Cu_2O$ NC absorbance indicated at 592 nm. (b) The corresponding Tauc Plot of FTO-$Cu_2O$ NC with the minimum value of absorbance taken as background. The bandgap is estimated to be 2.0 eV.

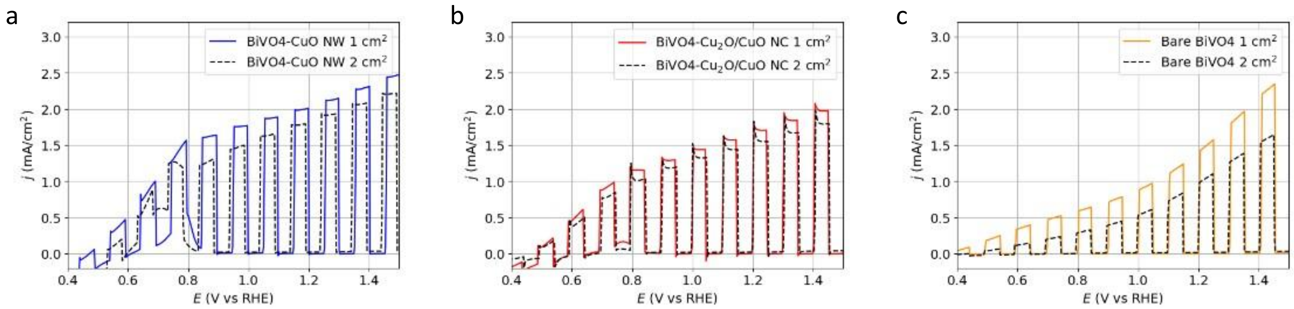


Figure S21: Different sample sizes: 1 $cm^2$ vs 2 $cm^2$ for a) $BiVO_4$-CuO NW, b) $BiVO_4$-$Cu_2O$/CuO NC, and c) bare $BiVO_4$. The scan rate was 10 mV/s.

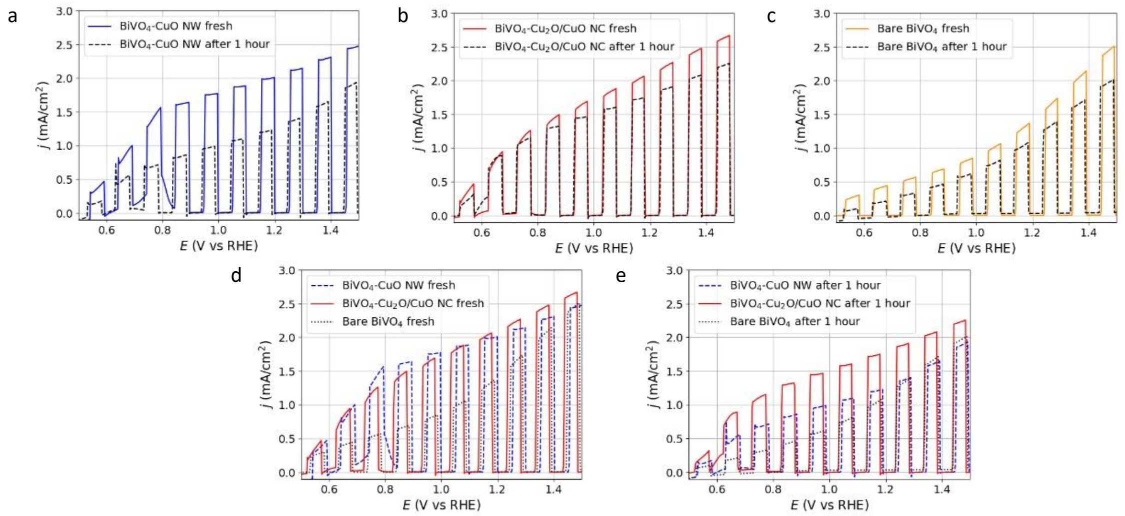


Figure S22: Comparison of LSV curves of before and after the chronoamperometry shown in Figure 4b, all measured in fresh electrolyte, for (a) $BiVO_4$-CuO NW, (b) $BiVO_4$-$Cu_2O$/CuO NC, and (c) bare $BiVO_4$. The same LSV curves displayed together (d) before and (e) after the one-hour chronoamperometry of Figure 4b, respectively. The scan rate was 10 mV/s.

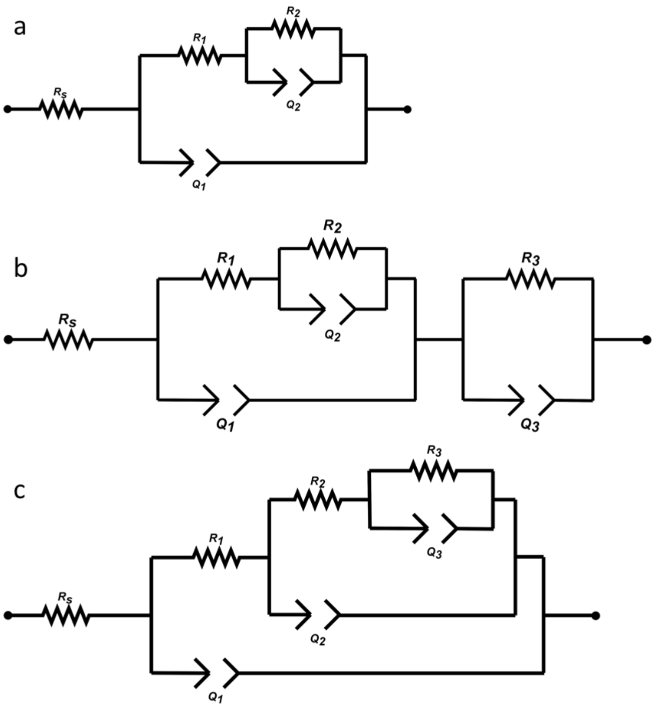


Figure S23: Models used to fit the EIS data. (a) double parallel circuit; (b) circuit extended with an additional element in series; (c) circuit extended with an additional element in parallel.[12]

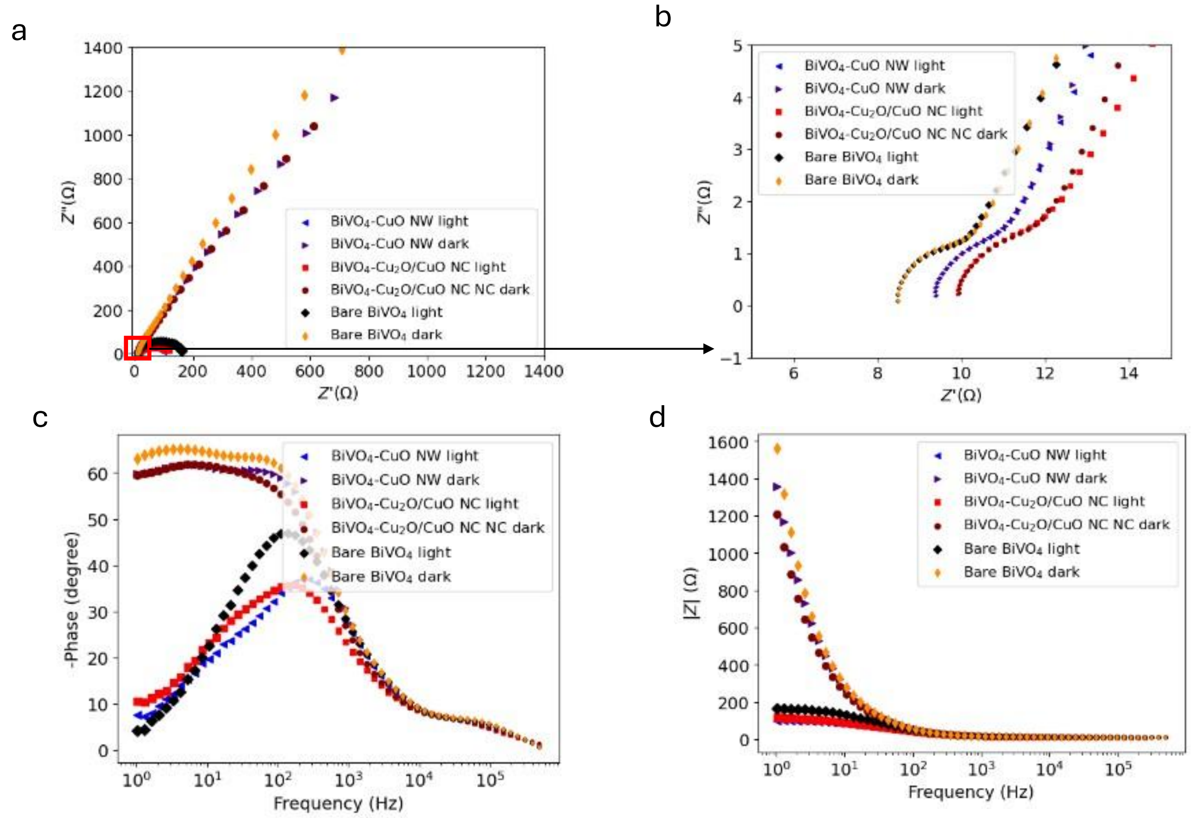


Figure S24: Comparison of EIS data in the light and dark. a) Nyquist plot of the different samples at 1.23V with and without illumination. b) Zoomed in Nyquist plot at low resistances c) Bode phase and d) Z plot of samples in the dark and in the light.[13]

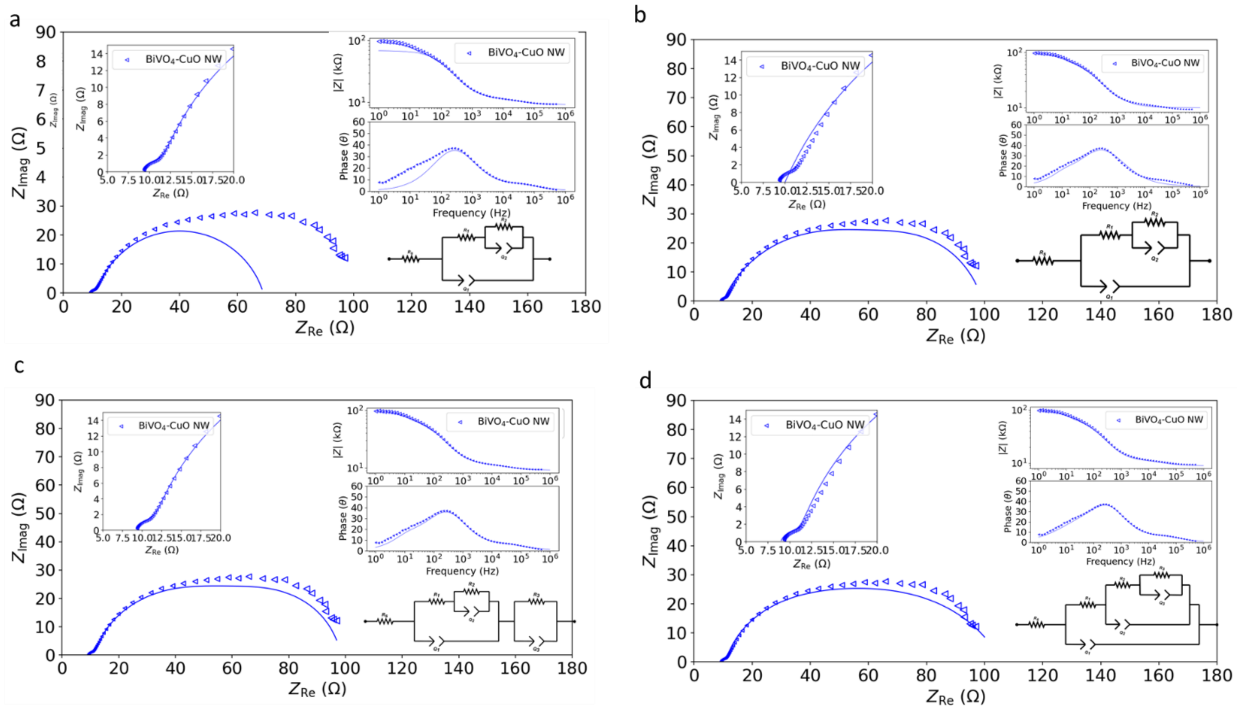


Figure S25: Possible fits of BiVO4-CuO NW EIS data, according to a) model S23a, b) Model S23a (with different parameters), c) Model S23b, d) Model S23c. Fitted parameters can be found in Table S2. Fits made with model S23a show significant deviations from the measured data in either the low frequency regio (a) or the high frequency region (b). A third circuit in series (c) or parallel (d) improves the fit, with the fit in series (c) yielding slightly better results (Table S3). [12,14]

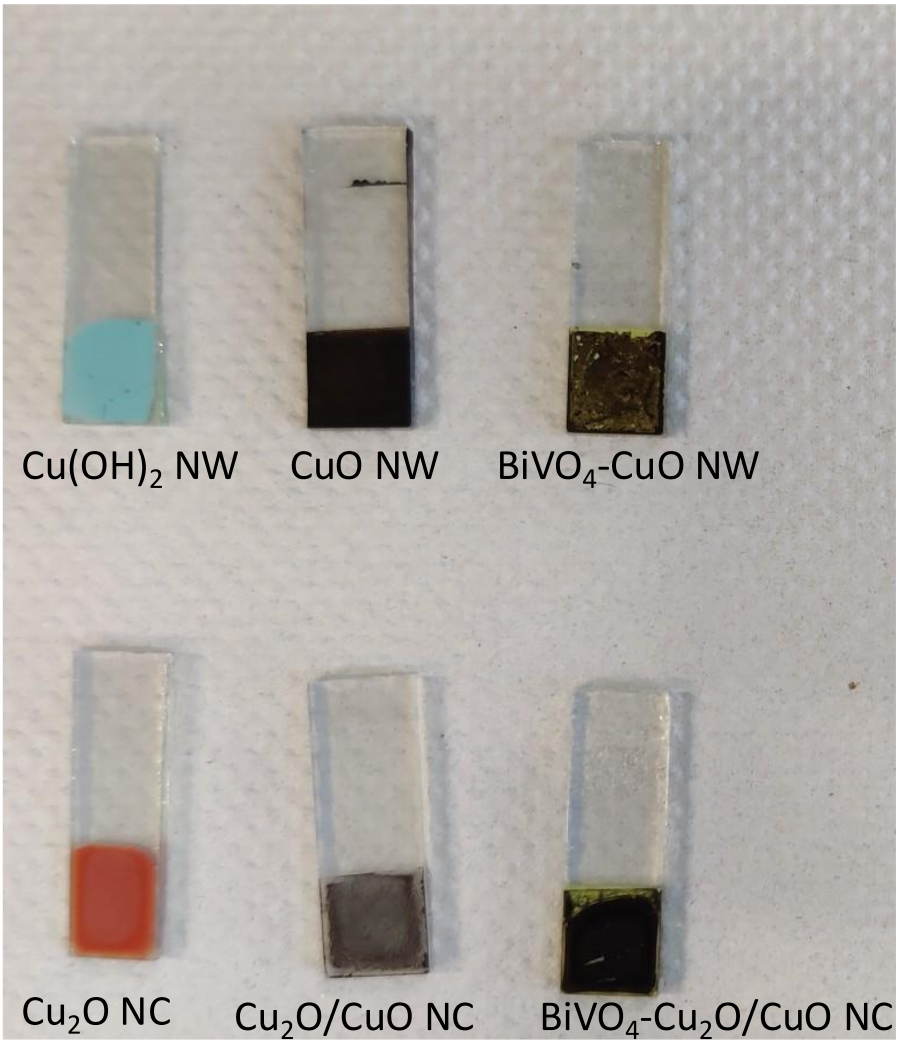


Figure S26: Photograph of $Cu(OH)_2$ NW, CuO NW, $BiVO_4$-CuO NW, $Cu_2O$ NC, $Cu_2O$/CuO NC and $BiVO_4$-$Cu_2O$/CuO NC samples on FTO taken before measurement.

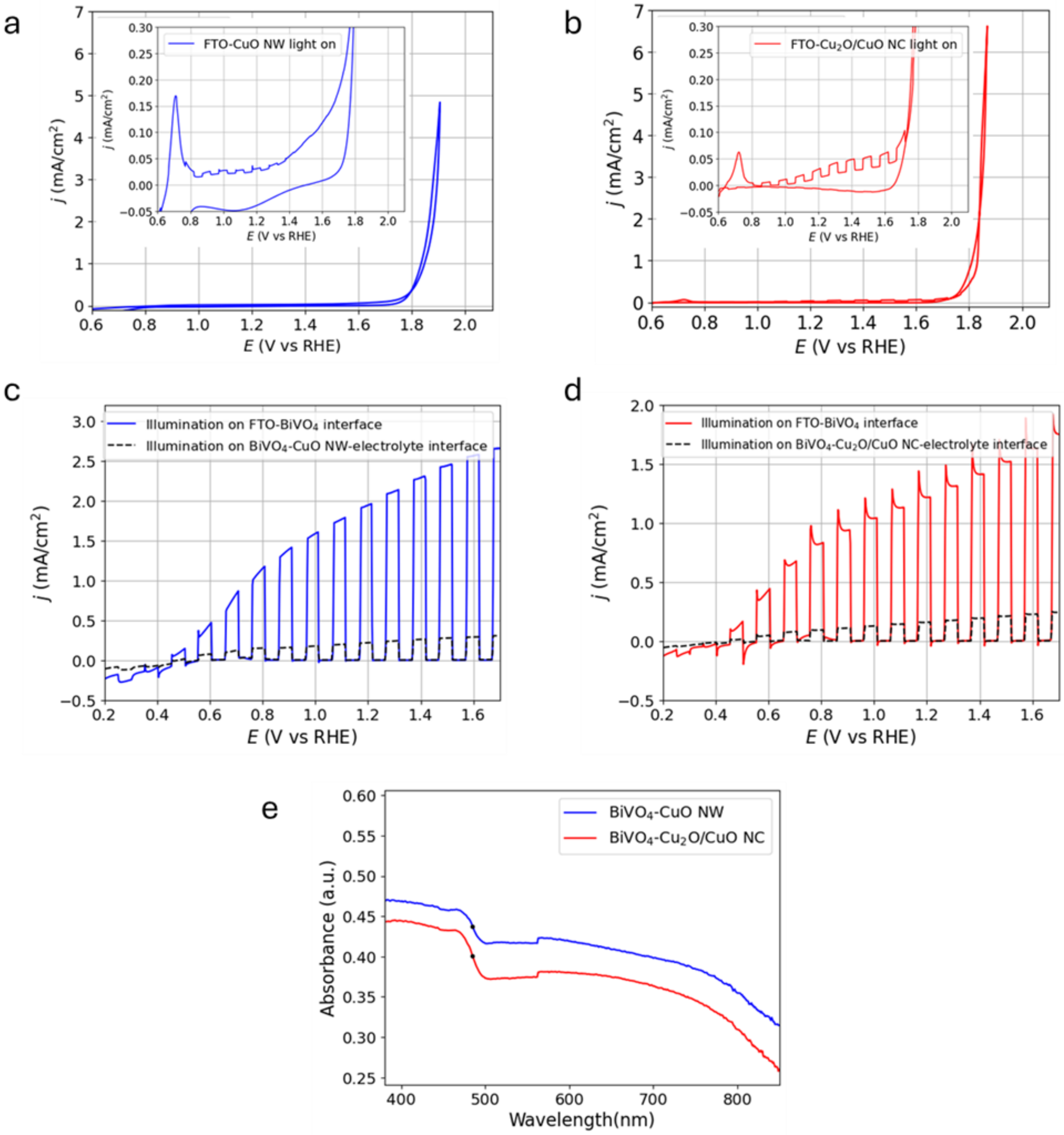


Figure S27: Light on/off plots for a) CuO NW and b) $Cu_2O$/CuO NC on FTO. A closeup is included. Difference between front and back illumination for c) $BiVO_4$-CuO NW and d) $BiVO_4$-$Cu_2O$/CuO NC. Scan speed was 10 mV/s. e) shows the UV-Vis absorbance of $BiVO_4$-$Cu_2O$/CuO NC and $BiVO_4$-CuO NW, with the midpoint of the reduction in absorbance indicated.

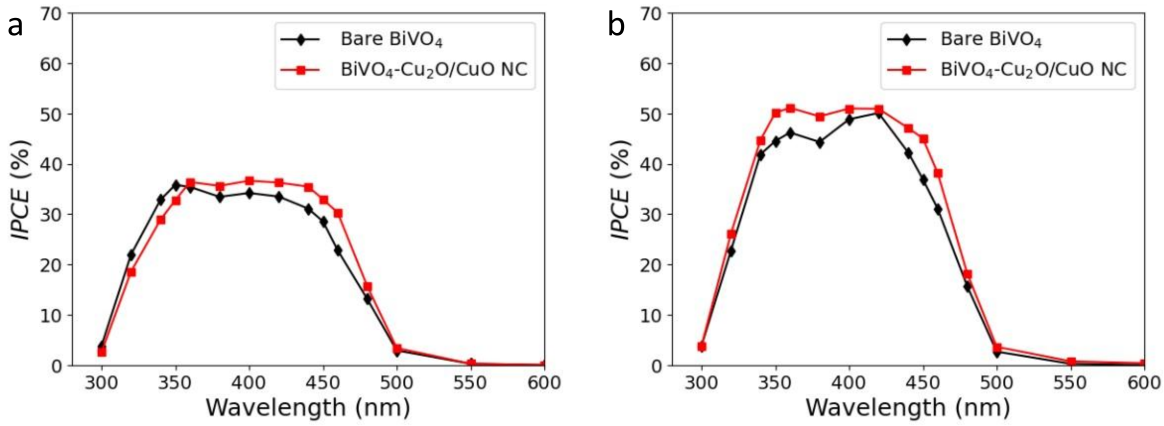


Figure S28: IPCE measurements for bare $BiVO_4$ and $BiVO_4$-$Cu_2O$/CuO NC in pH 9.5 1M KBi buffer with a) no hole scavenger and b) 1M $Na_2SO_3$ added as hole scavenger, calculated with eq 8. Measurements were performed at 1V vs RHE.

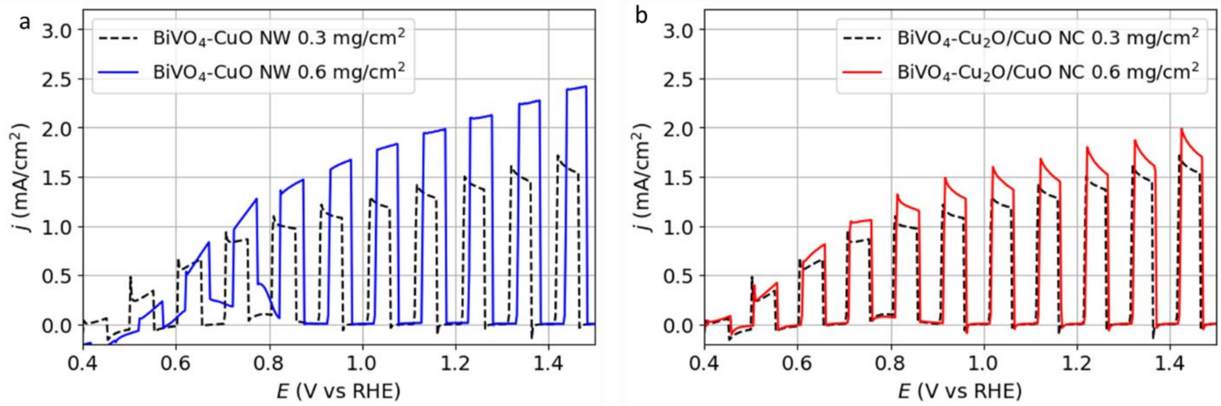


Figure S29: Light on/off plots for a) $BiVO_4$-CuO NW and b) $BiVO_4$-$Cu_2O$/CuO NC with 0.3 mg/cm$^2$ and 0.6 mg/cm$^2$ catalyst loading. Scan speed was 50 mV/s.

References


(1) Yang, Y.; Zhong, X.; Liu, K.; Du, J.; Yang, Y.; He, H.; Zhou, Y.; Dong, F.; Fu, C.; Wang, J. Insight into the Improvement Mechanism of Copper Oxide/BiVO 4 Heterojunction Photoanodes for Solar Water Oxidation. *Journal of The Electrochemical Society* **2019**, *166* (12), H513–H520. https://doi.org/10.1149/2.0611912jes.

(2) Meng, L.; Tian, W.; Wu, F.; Cao, F.; Li, L. TiO2 ALD Decorated CuO/BiVO4 p-n Heterojunction for Improved Photoelectrochemical Water Splitting. *Journal of Materials Science & Technology* **2019**, *35* (8), 1740–1746. https://doi.org/10.1016/j.jmst.2019.03.008.

(3) Murugan, C.; Pandikumar, A. Reinforcement of Visible-Light Harvesting and Charge-Transfer Dynamics of BiVO $_4$ Photoanode via Formation of p–n Heterojunction with CuO for Efficient Photoelectrocatalytic Water Splitting. *ACS Appl. Energy Mater.* **2022**, *5* (6), 6618–6632. https://doi.org/10.1021/acsaem.1c04120.

(4) Li, X.; Wan, J.; Ma, Y.; Wang, Y.; Li, X. Study on Cobalt-Phosphate (Co-Pi) Modified BiVO4/Cu2O Photoanode to Significantly Inhibit Photochemical Corrosion and Improve the Photoelectrochemical Performance. *Chemical Engineering Journal* **2021**, *404*, 127054. https://doi.org/10.1016/j.cej.2020.127054.

(5) Yang, L.; Wang, R.; Chu, D.; Chen, Z.; Zhong, F.; Xu, X.; Deng, C.; Yu, H.; Lv, J. BiVO4 Photoelectrodes for Unbiased Solar Water Splitting Devices Enabled by Electrodepositing of Cu2O Simultaneously as Photoanode and Photocathode. *Journal of Alloys and Compounds* **2023**, *945*, 169336. https://doi.org/10.1016/j.jallcom.2023.169336.

(6) Bai, S.; Han, J.; Zhao, Y.; Chu, H.; Wei, S.; Sun, J.; Sun, L.; Luo, R.; Li, D.; Chen, A. rGO Decorated BiVO4/Cu2O n-n Heterojunction Photoanode for Photoelectrochemical Water Splitting. *Renewable Energy* **2020**, *148*, 380–387. https://doi.org/10.1016/j.renene.2019.10.044.

(7) Bai, S.; Liu, J.; Cui, M.; Luo, R.; He, J.; Chen, A. Two-Step Electrodeposition to Fabricate the p–n Heterojunction of a $Cu_2$ O/$BiVO_4$ Photoanode for the Enhancement of Photoelectrochemical Water Splitting. *Dalton Trans.* **2018**, *47* (19), 6763–6771. https://doi.org/10.1039/C7DT04258B.

(8) *The International XPS Database of Monochromatic XPS Reference Spectra*. https://xpsdatabase.net/ (accessed 2025-09-11).

(9) Biesinger, M. C. Advanced Analysis of Copper X-ray Photoelectron Spectra. *Surface & Interface Analysis* **2017**, *49* (13), 1325–1334. https://doi.org/10.1002/sia.6239.

(10) Shao, P.; Deng, S.; Chen, J.; Chen, J.; Xu, N. Study of Field Emission, Electrical Transport, and Their Correlation of Individual Single CuO Nanowires. *Journal of Applied Physics* **2011**, *109* (2), 023710. https://doi.org/10.1063/1.3536478.

(11) Van Der Heijden, O.; Park, S.; Vos, R. E.; Eggebeen, J. J. J.; Koper, M. T. M. Tafel Slope Plot as a Tool to Analyze Electrocatalytic Reactions. *ACS Energy Lett.* **2024**, *9* (4), 1871–1879. https://doi.org/10.1021/acsenergylett.4c00266.

(12) Bredar, A. R. C.; Chown, A. L.; Burton, A. R.; Farnum, B. H. Electrochemical Impedance Spectroscopy of Metal Oxide Electrodes for Energy Applications. *ACS Appl. Energy Mater.* **2020**, *3* (1), 66–98. https://doi.org/10.1021/acsaem.9b01965.

(13) Mei, B.-A.; Munteshari, O.; Lau, J.; Dunn, B.; Pilon, L. Physical Interpretations of Nyquist Plots for EDLC Electrodes and Devices. *J. Phys. Chem. C* **2018**, *122* (1), 194–206. https://doi.org/10.1021/acs.jpcc.7b10582.

(14) Lazanas, A. Ch.; Prodromidis, M. I. Electrochemical Impedance Spectroscopy–A Tutorial. *ACS Meas. Sci. Au* **2023**, *3* (3), 162–193. https://doi.org/10.1021/acsmeasuresciau.2c00070.
